# A systematic review and meta-analysis of measurement properties of objective structured clinical examinations used in physical therapy licensure and a structured review of licensure practices in countries with well-developed regulation systems

**DOI:** 10.1371/journal.pone.0255696

**Published:** 2021-08-03

**Authors:** Pavlos Bobos, Dimitra V. Pouliopoulou, Alexandra Harriss, Jackie Sadi, Alison Rushton, Joy C. MacDermid

**Affiliations:** 1 School of Physical Therapy, Faculty of Health Science, Western University, London, ON, Canada; 2 Roth McFarlane Hand and Upper Limb Centre, St. Joseph’s Hospital, London, ON, Canada; 3 Department of Clinical Epidemiology and Health Care Research, Dalla Lana School of Public Health, Institute of Health Policy, Management and Evaluation, University of Toronto, Toronto, ON, Canada; 4 Applied Health Research Centre, Li Ka Shing Knowledge Institute of St. Michael’s Hospital, Toronto, ON, Canada; 5 Institute of Cardiovascular and Medical Sciences, University of Glasgow, Glasgow, United Kingdom; 6 Department of Hygiene, Epidemiology and Medical Statistics, National and Kapodistrian University of Athens, Athens, Greece; 7 School of Rehabilitation Science, McMaster University, Hamilton, ON, Canada; Xiamen University - Malaysia Campus: Xiamen University - Malaysia, MALAYSIA

## Abstract

**Background:**

The Objective Structured Clinical Examination (OSCE) is a commonly used tool internationally to assess clinical competency. Physical therapy (PT) licensure processes vary internationally. The OSCE is the tool used in Canada to assess clinical competency for PT graduates seeking licensure. Previous studies that examined the measurement properties of OSCEs present contradictory results.

**Objectives:**

The first objective was to investigate the reliability and validity of OSCEs when administered to PTs during their education or as part of a licensure process. The second objective was to conduct a structured review to report PT educational and licensing components and policies in 17 countries with well-developed PT regulation systems.

**Methods:**

An electronic search was performed in four databases from inception to 31^st^ March 2021 to identify relevant articles. Two reviewers performed the critical appraisal of the included studies using a validated quality assessment tool. We deployed a random effects meta-analysis on reliability and validity estimates of OSCEs and examined sources of heterogeneity with univariate meta-regressions. We searched websites of professional regulatory bodies and associations for data on educational and licencing components and policies. Educational and licensing components across countries were synthesized descriptively.

**Results:**

A pooled estimate of Cronbach’s alpha of 0.55, (95% CI: 0.41, 0.67) was determined for OSCEs. The pooled estimate of Intraclass Correlation Coefficient (ICC) between assessors was 0.77 (95% CI: 0.70, 0.83). The pooled estimate of Pearson Correlation between multiple OSCE stations’ scores was 0.27 (95% CI: 0.15, 0.39); and between each station score and the total score was 0.71 (95% CI: 0.61, 0.79). The pooled estimates for kappa Coefficients were 0.75 (95% CI: 0.58, 0.86) and 0.84, (95% CI: 0.72, 0.91) for intra-rater and inter-rater reliability of the standardised patient respectively. From the 17 included countries, Canada (excluding Quebec) was the only country that required both a clinical and written competency exam following graduation from an accredited PT program. Two countries (USA, UAE) required a written competency exam. The remaining 14 countries did not require an additional competency examination after completion of degree requirements from an accredited program.

**Conclusions:**

We found weak evidence that OSCE examinations items are internally consistent when used to assess PTs. Canada (excluding Quebec) is the only country out of 17 implementing a national clinical competency examination for their PT graduates to achieve licensure after completing professional degree requirements.

## 1. Introduction

In Canada, to obtain a licensure certification, physical therapists (PTs) must meet the standards of credentialing set out by the Canadian Alliance of Physiotherapy Regulators (CAPR). The CAPR administers the Physiotherapy Competency Exam, a national exam which consists of two parts, written and clinical, and it is independent of university educational programs (except from the province of Quebec) [1]. The evaluation tool used for the clinical component of the competency assessment in Canada is the Objective Structured Clinical Examination (OSCE) [2].

OSCEs are an interactive form of examination that are highly structured, incorporating case scenarios, uniform grading schemes, and standardized patients (SP) to provide a comprehensive evaluation of each student OSCEs are used globally across a variety of specialties [3–5] and evaluate clinical skills and judgement, to a greater extent than written tests. When OSCEs are used within educational programs, student performance can be considered in light of other aspects of their performance and remediation can be provided, before major decisions like removal from the program/professional would be made. The rationale and processes to implement OSCEs as a national licensure examination brings in additional issues and complications since the exams are administered by an independent body on a single occasion and decisions to prevent an individual from becoming a registered physical therapist are based on pass rules established by the examination regulators. Since the major role of regulators is protection of the public the extra effort to prevent incompetent professionals from entering the profession is important, and could justify the use of OSCEs if it improved protection of the public. If a licensure OSCE is justified, it is essential that the test accurately classifies competent and incompetent individuals.

Measurement properties of OSCEs are challenging to investigate as there are few reference standards and real-world testing cannot include test-retest reliability. Therefore, real-world evaluations tend to focus on easily achieved analyses like the correlation between stations (internal consistency) or comparison to other performance indicators (construct validity) that are accessible within the educational program. An exception to that is the test-retest reliability of assessment of performance through the standardised patient (SP), a measurement property that can be examined in real-world testing through repeated evaluations of the SP performance on the same scenario. Alternatively, simulations can be used, but may lack generalizability since the context and weight of the decision are not replicated. Another challenge is that OSCEs can vary in terms of the number of stations, observation times and numbers or training of assessors, and the structure can vary across different professions [6]. Thus, some variation in test performance may be related to the variations in the ways that the tests are constructed. The issue with using psychometric indicators that are easily achieved is that they may not address the most important reasons for using the examination. Internal consistency suggests concordance between scores across different stations of an OSCE, coming from either the same or different raters. The extent to which scores different stations should be related to each other is debatable. On the one hand we typically expect that people who are incompetent in one aspect of practice would be incompetent in other aspects of practice. However, this is not always the case. Further, comprehensive OCSEs are often designed to tap into very diverse aspects of practice, which would be expected to lower station internal consistency. More importantly, the primary function of the examination is to discriminate competent and incompetent applicants. Thus, discriminative validity is the most important concern. The real test of OSCEs is whether it can be depth’s traded that they provide additional protection to the public beyond that which is provided by the professional program evaluations. This is more difficult research to conduct and therefore psychometric studies often justify the need to look at a variety of psychometric evaluations.

While the value of a national OSCE-based entry-level to practice licensure exam has been debated prior to the pandemic, the issue has become urgent during the pandemic. Due to the COVID-19 pandemic, the OSCE examinations that lead to practice as a registered physical therapist have been paused for more than a year in Canada [7]. This has delayed the usual licensure process and prevented graduating physical therapist who have completed all the required elements of the professional training to be able to achieve timely licensure and could ultimately interfere public protection by reducing access to physical therapy. The Canadian Physiotherapy Association and the Colleges of Physiotherapists are looking into new ways and evidence to address this problem. Our goal was to facilitate their decision-making through a systematic synthesis of the psychometric evidence and a structured policy and standards review that would describe measurement properties of OSCEs in the profession of physical therapy and current practice for licensure examination.

### 1.1 Aims

1. To conduct a systematic review to investigate the measurement properties of OSCEs when administered to physical therapists during training or as part of a licensure process.
2. To conduct a structured review to report the physical therapy educational and licensing components and policies in 17 countries with well-developed regulation systems.

## 2. Materials and methods

### 2.1 Systematic review

A systematic review reported in line with the Preferred Reporting Items for Systematic reviews and Meta-Analyses (PRISMA). The steps that were followed were to identify the research question, to retrieve relevant studies, to chart the extracted data and synthesize and the results [8].

#### 2.1.1 Protocol and registration

A protocol was developed by the primary and senior author and the final version was approved from all the co-authors before the drafting of this manuscript. The final version of the study protocol is available at the **S1 Appendix**. We registered our protocol to the PROSPERO database (CRD42021253069).

#### 2.1.2 Objectives

The objective of the systematic review was to investigate the reliability and validity of OSCEs when administered to physical therapists during training or as part of a licensure process.

#### 2.1.3 Eligibility criteria

We included peer reviewed articles and pre-prints that reported reliability and validity statistics for OSCEs in the physical therapy profession without posing any language restriction.

#### 2.1.4 Search and information sources

An electronic search was designed in consultation with a research librarian with expertise in literature searching, and performed in 4 databases (PubMed, EMBASE, Google SCHOLAR and CINHAL) from inception to 31^st^ March 2021. A combination of search terms was used including “Clinical competence”, “Clinical performance”, “Workplace performance”,” “Summative assessment”, “Objective Structured Clinical Examination”, “OSCE”, “Reliability”, “Validity”, “Physiotherapy or Physical therapy” to identify relevant articles regarding the measurement properties of OSCEs. A grey literature search was also conducted through the Google web search engine. Reference lists were also manually searched from retrieved articles. The search strategy can be found in the **S1 Appendix**. We screened all the retrieved articles in duplicate (PB and DP) in 2 levels. An initial screen was performed through title and abstract and then a second screening at a full text level from 2 authors (PB and DP). If disagreements were present, they were resolved by a third author (JCM) through consensus.

#### 2.1.5 Data extraction and charting

Two authors (PB and DP) independently extracted and imported in duplicate all data from included studies into a web database (Covidence). The data extraction form was calibrated between the primary (PB) and senior author (JCM). For the peer reviewed and pre-print articles, we extracted information on author, year, country, OSCE, time duration, number of OSCE stations, number of examiners, sample size and reliability and validity statistics.

Taken from a previous study examining the reliability of a patient education performance tool in physical therapists, the benchmarks for Intraclass Correlation were quantified as “poor” (< 0.00), “slight” (0.00–0.20), “fair” (0.21–0.40), “moderate” (0.41–0.60), “substantial” (0.61–0.80), and “almost perfect” (> 0.80) [9]. According to a recent review that surveyed how the application of Cronbach’s alpha has been presented in major science education journals, most studies use the cut-off point of 0.7 to identify a score as “acceptable”, 0.7–0.8 is considered as “respectable” and 0.8–0.9 as “very good” [10]. Regarding Pearson and Spearman Correlation, the guide presented by Evans was utilized to quantify the strength of the correlation as follow; 0.00–0.19 “very weak”, 0.20–0.39 “weak”, 0.40–0.59 “moderate”, 0.60–0.79 “strong”, 0.80–1.00 “very strong” [11]. As for kappa Coefficient, Cohen suggested the Kappa result be interpreted as follows: values ≤ 0 as indicating no agreement and 0.01–0.20 as none to slight, 0.21–0.40 as fair, 0.41–0.60 as moderate, 0.61–0.80 as substantial, and 0.81–1.00 as almost perfect agreement [12].

#### 2.1.6 Critical appraisal of the included studies

A summary score for the overall quality of individual studies was appraised independently by the authors (PB) and (DP) using a structured clinical measurement specific appraisal tool [13, 14]. In case of disagreement a third person was consulted (JCM) to resolve the conflict. The evaluation criteria of this tool included twelve items: 1) Thorough literature review to define the research question; 2) Specific inclusion/exclusion criteria; 3) Specific hypotheses; 4) Appropriate scope of psychometric properties; 5) Sample size; 6) Follow-up; 7) The authors referenced specific procedures for administration, scoring, and interpretation of procedures; 8) Measurement techniques were standardized; 9) Data were presented for each hypothesis; 10) Appropriate statistics-point estimates; 11) Appropriate statistical error estimates; and 12) Valid conclusions and recommendations [13]. An article’s total score–quality—was calculated by the sum of scores for each item, divided by the numbers of items and multiplied by 100% [13]. Overall, the quality summary of appraised articles range from (0%-30%) “poor”, (31%-50%) “fair”, (51%-70%) “good”, (71%-90%) “very good”, and (>90%) “excellent” [13].

#### 2.1.7 Synthesis of results

Meta-analyses of reliability and validity coefficients were performed using STATA (StataCorp. 2019. Stata Statistical Software: Release 16. College Station, TX: StataCorp LLC) with “meta” package. We deployed a random effects maximum likelihood model. The extracted coefficients were converted to z values. Heterogeneity was deemed substantial if I^2^ values were greater than 50%, using the benchmarks of >70% for “high” heterogeneity, 50%-70% for “moderate” and <50% for “low” heterogeneity [15]. Forest plots were created using 95% CIs for coefficient estimates. We performed univariate meta-regressions if statistical heterogeneity was deemed substantial on pre-specified covariates such as Country and field of OSCE (e.g., musculoskeletal). Publication bias was evaluated through funnel plots [16]. In the presence of publication bias, we planned to impute the missing studies to account for publication bias in the meta-analysis. We compared the observed and the imputed studies vs observed only by using the non-parametric “trim and fill” method [17].

### 2.2 Structured review

#### 2.2.1 Objectives

The first objective of the structured review was to investigate and report the educational and licensing components and policies of the physical therapy profession in countries with a well-developed regulation system around the world. The second objective was to investigate and report the ongoing competency assessments used for licensure renewals and performance controls.

#### 2.2.2 Eligibility criteria

To generate a broad picture of the types of education and professional quality assurance systems implemented on a worldwide scale, a selective global scan of 17 countries was conducted. The selection was primary based on best-available, systematically recorded, online accessible data. The inclusion criteria were countries where the physical therapy profession is regulated. Countries were excluded if the physical therapy profession was in existence for less than 20 years, or if the member base included less than 1 physical therapist per 10,000 population. The proportion of physical therapists per 10,000 population for each country selected can be seen in **S1 Appendix.**

#### 2.2.3 Search and information sources

Due the nature of the structured review, the basic source of information was grey literature derived from websites of professional regulatory bodies and associations. These websites were searched throughout for information regarding education and license requirements and quality assurance methods. To gather as much information as possible, apart from the initial webpage, up-to-date external links on program reports, presentations, law and regulation documents, applicant instructions documents and application forms the were also searched.

#### 2.2.4 Data charting

Information extracted for the licencing components and policies in the different countries included minimum years of education, minimum supervised clinical hours in the curriculum, competency examination and licensure policy for domestic and international candidates, passing scores, passing rates, retakes policy, jurisprudence examination policy, license renewal policy, number of years to renewal, minimum continuing professional development hours and audit policy.

#### 2.2.5 Synthesis of results

Data for the licencing components and policies from different countries were synthesized with descriptive analysis by reporting frequencies and percentages for home graduates and for international applicants.

## 3. Results

### 3.1 Systematic review

**Fig 1:**
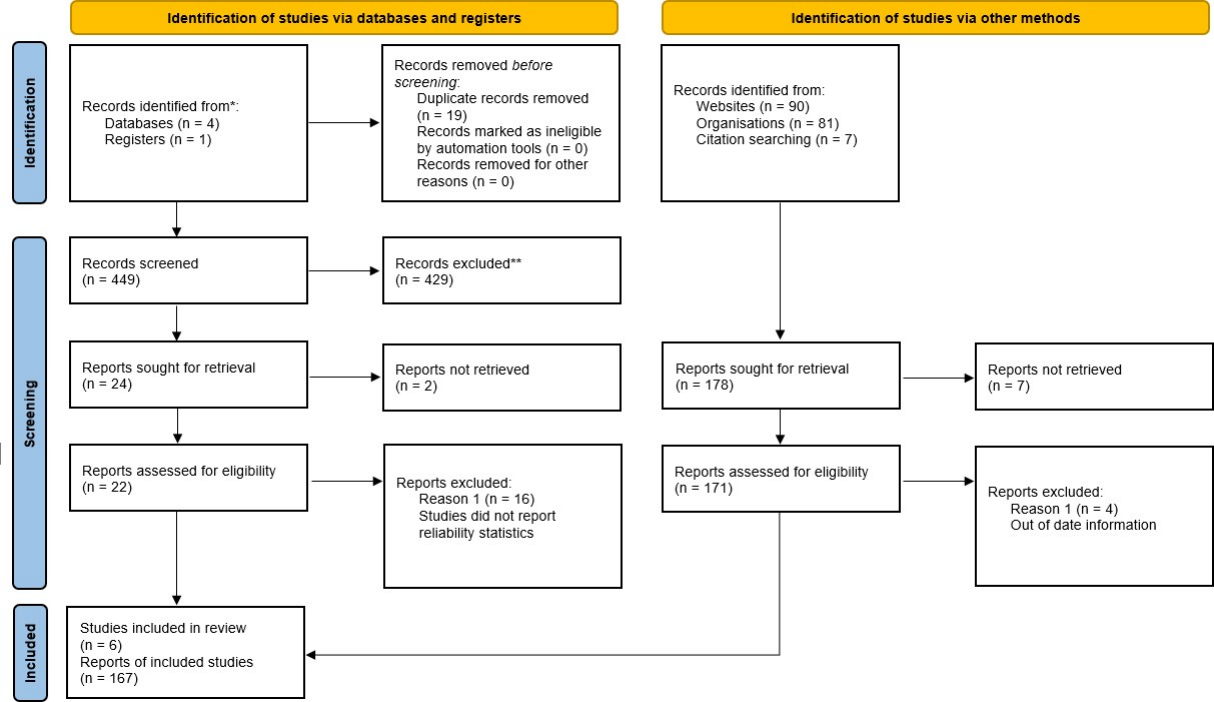
PRISMA flow chart.

From our initial electronic search we identified, 449 articles. After removal of duplicates, 429 articles were excluded through title and abstract review and 25 were deemed relevant for a full text review. Of the 25 studies that were deemed relevant, two reports could not be retrieved and 6 studies met our inclusion criteria and were included in the analysis (**Fig 1**).

Through grey literature search we identified one dissertation [18] reporting on data deriving from the same study as one of our peer reviewed articles i.e. 2 citations for 1 study [19]. We only included the peer reviewed article in the main statistical analysis to avoid inserting duplicated information and conducted sensitivity analyses including the dissertation where additional data were available.

#### 3.1.1 Characteristics of included studies

From the six included studies, two were conducted in Canada [20, 21], one in Brazil [22], one in Australia [23] and two were conducted in the USA [6, 18, 19]. All studies were conducted in a university research unit. The majority of the studies (60%) included post-graduate physical therapy students [6, 18–20]. One study included undergraduate physical therapy students [22], one study did not specify whether the students were undergraduates or postgraduates [21], and one study used a mix of undergraduate physical therapy students and practicing physical therapists [23]. The mean number of participants across studies was 47, with a minimum of 16 and a maximum at 66 participants. Three studies used data from real world examinations (observational) [6, 18–20] and three designed a simulated examination to fit the purposes of the study (experimental [21–23]. None of the studies was conducted for licensure purpose [6, 19–23]. Five studies used a cross-sectional design to evaluate the student’s performance [6, 19–22], and one performed repeated measurements (longitudinal) evaluating the SP performance [23].

*3*.*1*.*1*.*1 Type of OSCEs*. Four studies used a musculoskeletal (MSK) OSCE format [18–21, 23], one study used a Respiratory Physical Therapy format [22] and one study used a Neuromuscular version [6]. All studies consisted of two groups of participants, and one study had a cross over design [22]. Most of the studies used SP across all OSCE stations [18, 19, 21–23]. In four out of the six studies a different SP was used for each station [6, 20, 22, 23], while two studies used the same SP across multiple stations [18, 19, 21]. Two studies also included some non-interactive written stations [6, 20].

*3*.*1*.*1*.*2 Duration of OSCEs*. The timing of each station varied between studies. In Wessel 2002, each candidate was given five minutes per station [20]. Silva et al. 2011 gave each participant six minutes [22], and Swift et al. 2013 gave a total of 35 minutes to complete six stations [18, 19]. Gorman et al. 2010 gave each participant 45 minutes per station [6]. The rest of the studies did not put a time limit to the stations [21, 23]. Similarly, the number of the stations varied between studies. One study used a nine-station format [21]. Two studies included eight stations per participant [6, 20] and the rest of them included seven [18], six [19] and five [22] stations. One study used a single station OSCE format [23]. All stations had to be completed in one day, apart from the second group of participants in Gorman 2010 [6] that completed four stations on the first day and another four on the following day.

*3*.*1*.*1*.*3 Assessors in OSCEs*. Three studies used a single assessor per station [18–20, 22], but in one of them the process was recorded and two external examiners were asked to grade the examination independently [18, 19]. Two studies used two assessors per station [20, 23]. One study did not clearly specify the number of assessors per station [6].

*3*.*1*.*1*.*4 Reliability and internal consistency measurements*. Four studies reported a Cronbach’s alpha as a measurement for internal consistency between station scores [6, 18–20, 22]. To measure agreement between different examiners on the same stations two studies reported Intraclass Correlation Coefficient (ICC) [18, 19, 21]. One of them also reported the confidence intervals between different examiner’s grading [18, 19]. One study performed an Anova analysis. The researchers assigned the students randomly to one of two 8-station circuits. The stations in one circuit were identical to the stations in the other except for the examiner and the person playing the standardized patient role. A two-way analysis of variance (ANOVA) was used to determine differences among stations and between circuits. The authors hypothesized that if no difference was found between circuits, that would support the reliability of the assessors and the SP, whereas if no difference was found between station that would support the internal consistency of this OSCE [20].

Two studies performed a Pearson correlation of scores across different stations one comparing the scores of five [20] and the other the scores of four [6] stations. The latter also calculated a Pearson’s Correlation score comparing each station’s score to the total examination score.

One study reported on the simulated patient’s inter-rater and intra-rater correlation using kappa Coefficient [23]. This was the only study with a longitudinal design. The SP performance was measured by whether the essential clinical features were presented correctly during each encounter or not. This was assessed by an investigator that directly observed and recorded each encounter and filled a checklist on patient history and physical examination criteria using a 3-point scale of “not done”, “done poorly/incompletely”, “done well”. The process was repeated through a total of 12 encounters. The investigator’s ratings were used to calculate the interrater reliability of the SP. The SP was also asked to fill the same checklist after each encounter as a form of self-evaluation. Two weeks later the SP was asked to observe half of the original encounters on tape and re-evaluate himself using the same checklist. The Kappa coefficient between the original SP ratings and the SP ratings two weeks later were used to calculate the SP’s intrarater reliability.

*3*.*1*.*1*.*5 Validity measurements*. In terms of validity measurements four studies investigated the construct validity of OSCEs [6, 20, 22, 23]. One study reported Pearson correlations between the individual’s OSCE score to their final course score and their total GPA score [6]. One study reported Pearson correlations comparing an OSCE examination score to the score in a non-OSCE examination. Both examinations were simulated ones that were created to fit the needs of the study [22]. One study reported Spearman’s Correlation between the OSCE score and the total performance score during Supervised Clinical Practice [20]. One used an effect size analysis and an independent sample t test to estimate the known groups construct validity comparing two groups of participants with different knowledge level (practicing physical therapists with physical therapy students) [23]. The characteristics of the included studies are summarized in Table 1.

**Table 1:** Study characteristics.

Author (year)	Country	Setting	Study Design	Sample size	OSCE field	Educational level of participants	Number of OSCEs assessed	OSCE duration (min)	Number of assessors at each OSCE	Study Objective	Standardise Patients characteristics	Assessors’ characteristics	Purpose	Type of exams
Swift 2013	USA	Kansas Medical Center (KUMC) & Rockhurst University (RU)	Cross-sectional	65	MSK	Postgrad PT students	6	35(total)	3	Reliability	8 upper class PT students (2h training—multiple stations)	6 clinicians with experience in orthopaedic physical therapy	Low-stake purpose	Real life exam
Silva 2011	Brazil	Public Universidade de São Paulo (USP)	Cross-sectional	47	Chest	Undergrad PT Students	5	6 (per station)	1	Reliability & Validity	Professionals with least five years of experience	Professionals with least five years of experience	Low-stake purpose	Simulation exam
Gorman 2010	USA	Samuel Merritt University, inpatient setting	Cross-sectional	66	Neuro-MSK	Postgrad PT students	8 (4 with Standardised Patients)	45 (per station)	NA	Reliability & Validity	Trained health care providers (PTs, OTs & 1 experienced Nurse, one per station)	NA	Low-stake purpose	Real life exam
Wessel 2002	Canada	Mc Master University	Cross-sectional	48	MSK	Postgrad PT students	8 (5 with Standardised Patients)	5 (per station)	1	Reliability & Validity	Trained Standardised or real patients (one per station)	Practicing PTs trained in station-pairs	Low-stake purpose	Real life exam
Ladyshewsky 2000	Australia	Curtin University of technology	Longitudinal	16	MSK	Undergrad PT Students & PTs	1	NA	2	Reliability & Validity	One trained Standardised patient	Actor (30h training)	Low-stake purpose	Simulation exam
Stratford 1990	Canada	Mohawk College	Cross-sectional	24	MSK	PT students	9	NA	2	Reliability	One of the raters acted as the Standardised patient (multiple stations)	8 clinical education coordinators (30min training)	Low-stake purpose	Simulation exam

#### 3.1.2 Critical appraisal of the included studies

The quality of the included studies ranged between good (58.3%) to very good (87.5%) (**Table 2**). The most common flaws were 1) lack of/inadequate sample size calculations, and 2) missing data (i.e. inadequate follow up).

**Table 2:** Quality appraisal of the included studies.

Study	Item Evaluation Criteria*													
	1	2	3	4	5	6	7	8	9	10	11	12	Total (%)	Quality Summary
Swift et al (2013)	2	2	2	1	0	NA	2	2	2	2	1	1	77.2	Very Good
Wessel et al. (2002)	2	1	2	1	0	NA	2	2	1	1	0	1	59.0	Good
Silva et al (2011)	2	2	2	1	0	NA	2	2	2	2	2	2	86.4	Very Good
Gorman et al (2010)	2	2	2	2	0	NA	2	2	2	2	2	2	90.1	Excellent
Swift et al. (2007)	2	2	2	1	0	NA	2	2	2	2	2	2	86.4.5	Very Good
Ladyshewsky et al. 2000	2	2	2	2	0	NA	1	2	1	2	2	2	81.8	Very Good
Stratford et al. 1990	2	2	2	2	0	NA	1	2	2	2	2	2	95.5	Excellent

*Item Evaluation Criteria: 1. Thorough literature review to define the research question; 2. Specific inclusion/exclusion criteria; 3. Specific hypotheses; 4. Appropriate scope of psychometric properties; 5. Sample size; 6. Follow-up; 7. The authors referenced specific procedures for administration, scoring, and interpretation of procedures; 8. Measurement techniques were standardized; 9. Data were presented for each hypothesis; 10. Appropriate statistics-point estimates; 11. Appropriate statistical error estimates; 12. Valid conclusions and clinical recommendations.

Total score = (sum of subtotals ÷ 24 × 100). If for a specific paper an item is deemed NA (Not Applicable), then, Total score = (sum of subtotals ÷ (2 × number of Applicable items) × 100).

NA–Not Applicable. The subsections no. 6, asks for percentage of retention/follow up. This subsection only applies to reliability test-retest studies

Quality Summary: Poor (0%-30%), Fair (31%-50%), Good (51%-70%), Very good (71%-90%), Excellent (>90%)

#### 3.1.3 Reliability and internal consistency pooled estimates

**Fig 2:**
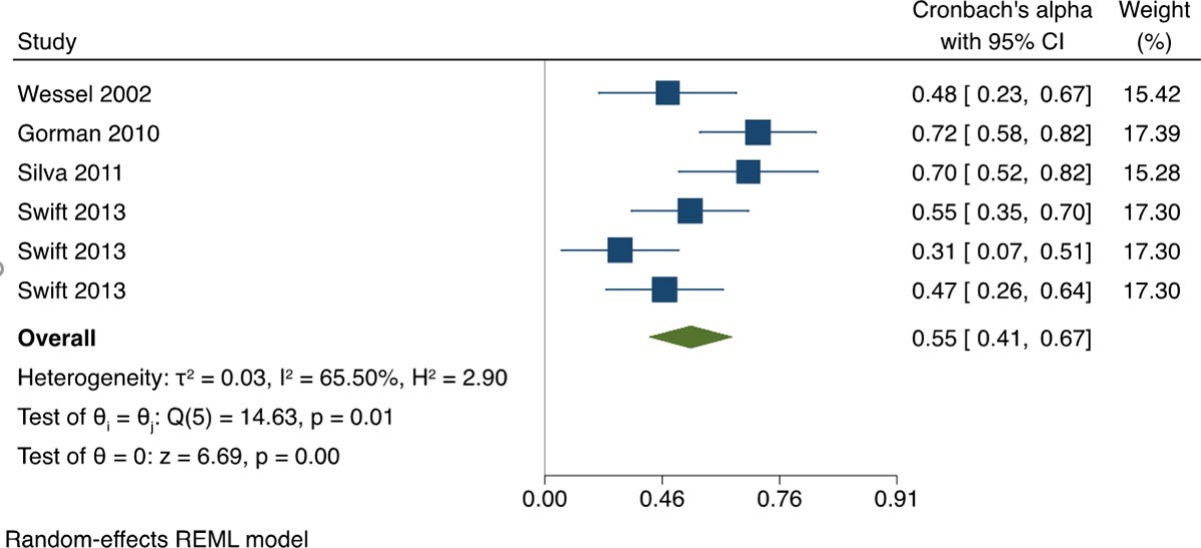
Meta-analysis of Cronbach’s alpha on OSCEs in physical therapy students. Forest plot presenting the meta-analyzed estimate on Cronbach’s alpha. Each square presents the results of an individual study with the size of the square being proportional to the weights used in the meta-analysis and the horizontal lines indicating the 95% confidence intervals. The solid vertical line represents no reliability, and the solid diamond indicates the overall summary measure.

**Fig 3:**
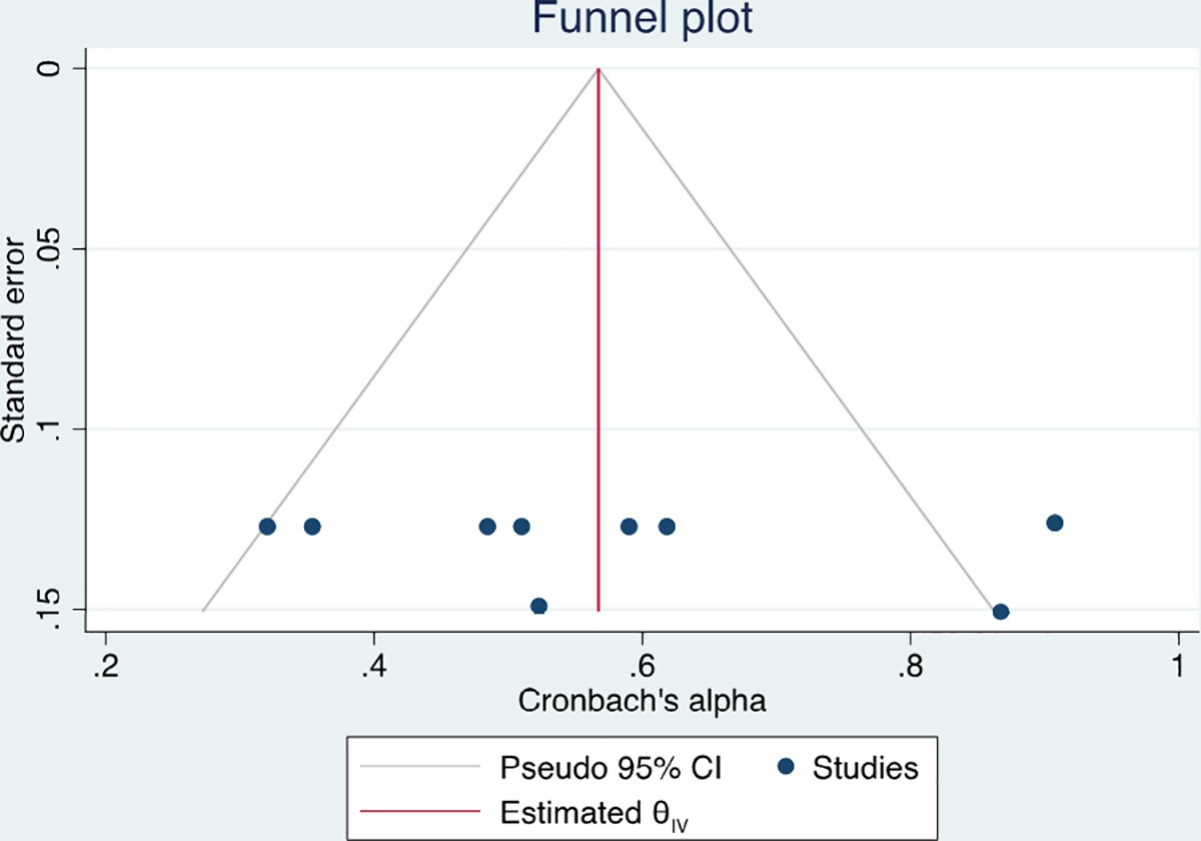
Funnel plot to for publication bias. Funnel plot presenting the studies alpha values from OSCEs, were each circle represents a study.

*3*.*1*.*3*.*1 Internal consistency between OSCE score elements (Cronbach’s alpha)*. Based on the available data, we found a pooled estimate of Cronbach’s alpha between OSCE station scores of 0.55, (95% CI: 0.41, 0.67) with moderate statistical heterogeneity, I^2^ = 65.5% (**Fig 2**). This alpha value is considered moderate, slightly below the acceptable range. A univariate meta-regression was conducted to investigate the sources of the moderate statistical heterogeneity. The regression coefficient for the field of OSCE was statistically significant p<0.0001, (0.25, 95% CI: 0.10, 0.39) with 98% of between-study variance explained (R^2^ = 97.85%). The funnel plot did not indicate presence of publication bias (**Fig 3**). A sensitivity analysis including Swift et al. 2007 [18] in Cronbach’s alpha estimates is available in **S1 Appendix.**

**Fig 4:**
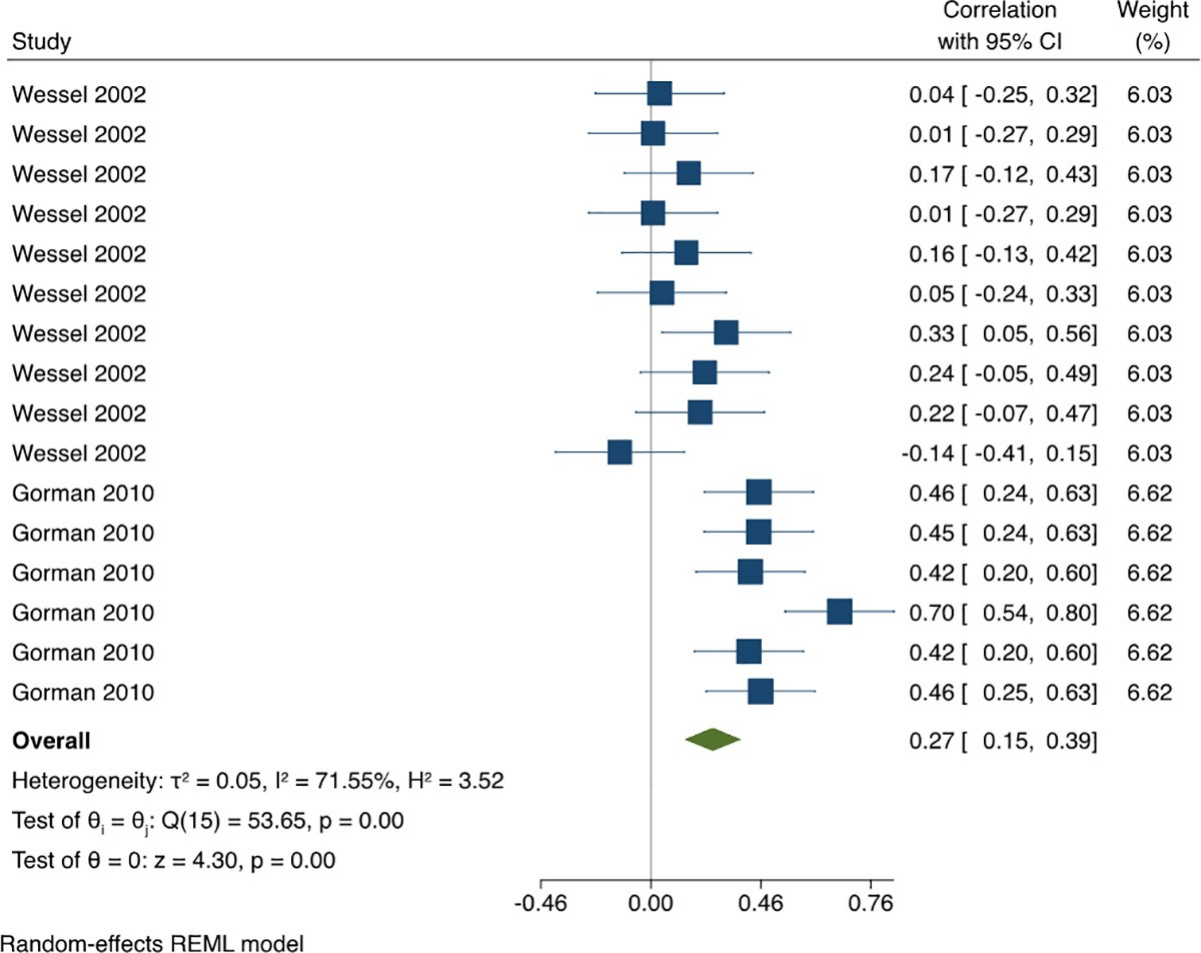
Meta-analysis of Pearson correlation between OSCEs stations in physical therapy students. Forest plot presenting the meta-analyzed estimate on Pearson’s r. Each square presents the results of an individual study with the size of the square being proportional to the weights used in the meta-analysis and the horizontal lines indicating the 95% confidence intervals. The solid vertical line represents no reliability, and the solid diamond indicates the overall summary measure.

**Fig 5:**
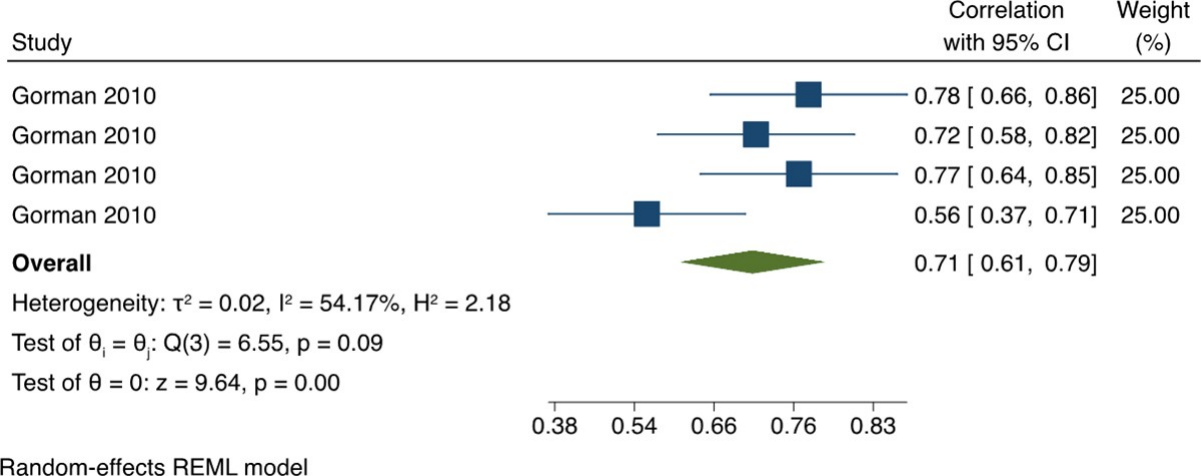
Meta-analysis of Pearson correlation between individual OSCEs stations’ score and total score in physical therapy students. Forest plot presenting the meta-analyzed estimate on Pearson’s r. Each square presents the results of an individual study with the size of the square being proportional to the weights used in the meta-analysis and the horizontal lines indicating the 95% confidence intervals. The solid diamond indicates the overall summary measure.

*3*.*1*.*3*.*2 Association between OSCE score elements (Pearson correlation coefficients)*. The pooled estimate of Pearson’s correlation in between station score correlation was 0.27 (95% CI: 0.15, 0.39) with high statistical heterogeneity, I^2^ = 71.55% (Fig 4). A univariate meta-regression was conducted to investigate the sources of the high statistical heterogeneity. The regression coefficient for the field of OSCE was statistically significant p<0.0001, (0.43, 95% CI: 0.28, 0.58) with 93% of between-study variance explained (R^2^ = 92.85%). The pooled estimate of Pearson Correlation between each station score and the total score based on all the available data was 0.71 (95% CI: 0.61, 0.79) with moderate statistical heterogeneity, I^2^ = 54.17% (Fig 5). This indicated a weak correlation between station’s score and a strong correlation between each station’s score and total OSCE score.

**Fig 6:**
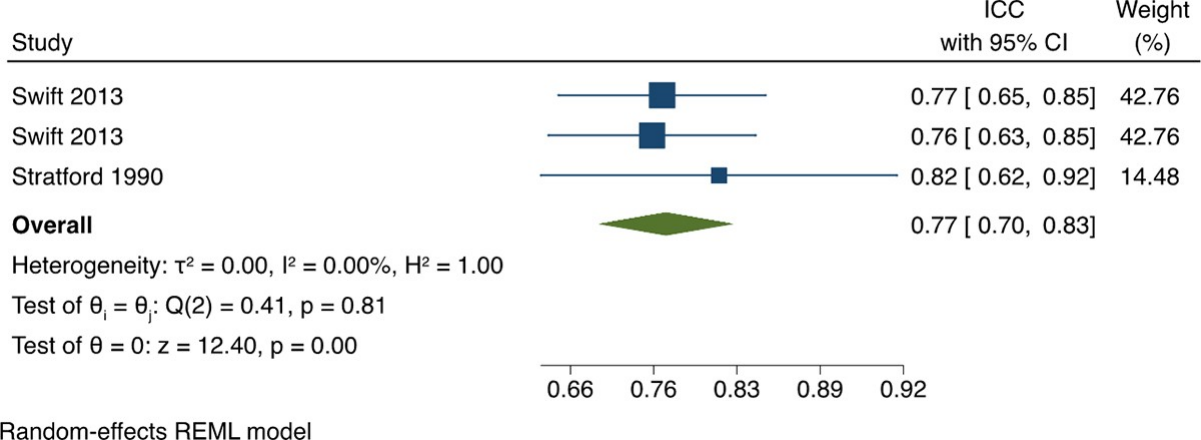
Meta-analysis of intraclass correlation coefficient (ICC) on OSCEs in physical therapy students. Forest plot presenting the meta-analyzed estimate on ICC. Each square presents the results of an individual study with the size of the square being proportional to the weights used in the meta-analysis and the horizontal lines indicating the 95% confidence intervals. The solid diamond indicates the overall summary measure.

*3*.*1*.*3*.*3 Intra-rater agreement between OSCE assessor elements (intra-class correlation coefficients)*. The pooled estimate of Intra-class Correlation Coefficient (ICC) between assessors based on the available data was 0.77 (95% CI: 0.7, 0.83) with no statistical heterogeneity, I^2^ = 0% (Fig 6). This indicated a substantial correlation in terms of interrater reliability. A sensitivity analysis including Swift et al. 2007 [18] in ICC estimates is available in S1 Appendix.

**Fig 7:**
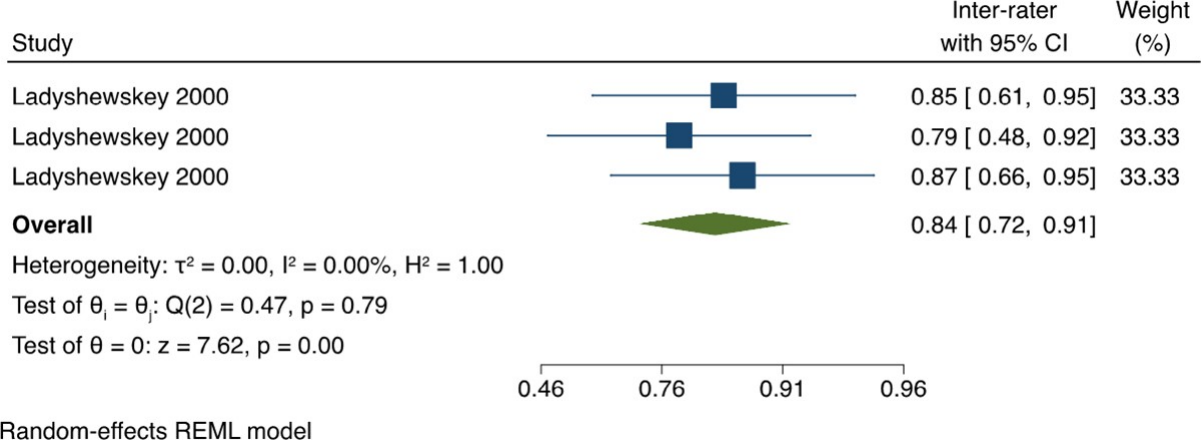
Meta-analysis of kappa coefficient for inter-rater reliability between standardised patients on OSCE’s in physical therapy students. Forest plot presenting the meta-analyzed estimate on kappa coefficient. Each square presents the results of an individual study with the size of the square being proportional to the weights used in the meta-analysis and the horizontal lines indicating the 95% confidence intervals. The solid diamond indicates the overall summary measure.

**Fig 8:**
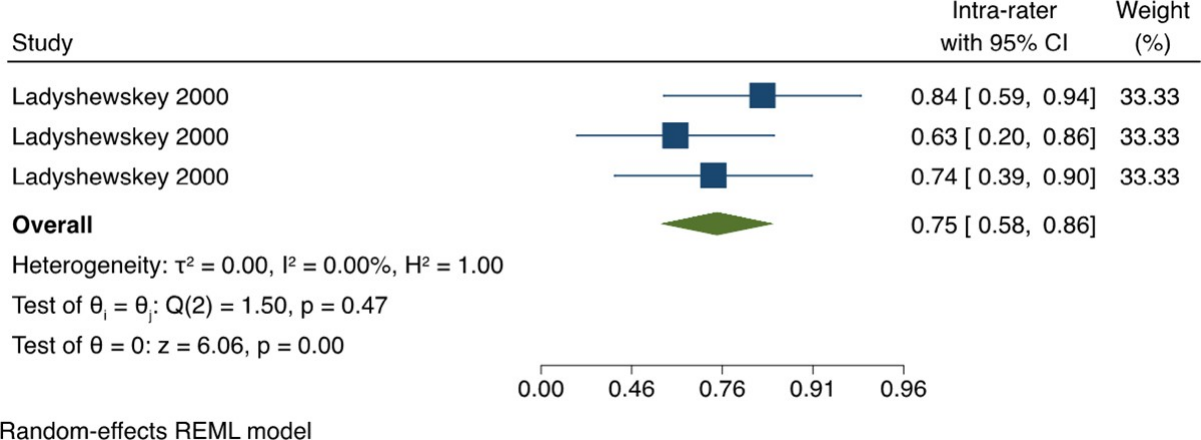
Meta-analysis of kappa coefficient for intra-rater reliability between standardised patients on OSCE’s in physical therapy students. Forest plot presenting the meta-analyzed estimate on kappa coefficient. Each square presents the results of an individual study with the size of the square being proportional to the weights used in the meta-analysis and the horizontal lines indicating the 95% confidence intervals. The solid diamond indicates the overall summary measure.

*3*.*1*.*3*.*4 Classification agreement between SP performance (kappa)*. For the inter-rater reliability between SP, the pooled estimate of kappa Coefficient based on all the available data was 0.84 (95% CI: 0.72, 0.91) with no statistical heterogeneity, I^2^ = 0% (Fig 7). For the intra-rater reliability between SP, the pooled estimate of kappa Coefficient was 0.75 (95% CI: 0.58, 0.86) with no presence of statistical heterogeneity, I^2^ = 0% (Fig 8). This indicates an almost perfect agreement regarding the investigator’s ratings on the SP performance, and a substantial agreement regarding the self-evaluation ratings done by the SP

#### 3.1.4 Non–pooled reliability estimates of the physical therapy OSCEs

The rest of the reliability estimates presented with high heterogeneity among studies and therefore, were reported in a non-pooled analysis. Swift et al. 2013 [19] reported the 95% Confidence Intervals of the pairwise differences between examiners in mean changes that were (-7.77, 4.01), (-11.98, - 0.18) and (-10.08, 1.71) for Examiner 1/ Examiner 2, Examiner 1/Examiner 3 and Examiner 2 / Examiner 3 respectively. Wessel et al. 2002 performed a two-way analysis of variance (ANOVA) with repeated measures to determine differences among stations and between groups of participants [20]. The ANOVA revealed significant differences among stations (F = 62.6, p < 0.001) but not between groups (F = 1.8, p = 0.19). There was no significant interaction between group and station (F = 1.1, p = 0.36).

#### 3.1.5 Validity estimates of the physical therapy OSCEs

Wessel et al. 2002 [20] used Spearman Rank correlation (ρ) to describe the association between 48 postgrad physical therapy students’ scores across an eight-station real life OSCE examination and their performance scores obtained during supervised clinical practice and found a very weak correlation of ρ = -0.13. Gorman et al. 2010 [6] compared 66 postgrad physical therapy students’ OSCE score across an eight-station format of a real-life course examination, to their final grade in stated course and to their total GPAs and found a Pearson’s correlation of 0.78 indicating very strong correlation and a Pearson’s correlation of 0.44 indicating moderate correlation respectively. Silva et al. 2011 [22] compared the six-station simulated OSCE examination scores of 47 undergrad students with their scores of a traditional exam including four theoretical and one practical question. Pearson’s correlation was = -0.1 indicating a very weak correlation. Finally, Ladyshewsky et al. 2000 [23] used an effect size analysis and an independent sample t test to estimate the construct validity of a single station simulated OSCE examination. They compared the OSCE scores of 12 Undergrad physical therapy students to those of 4 practising physical therapists and hypothesized that if the examination process was valid, practising physical therapists would perform better than physical therapy students. The effect size was 1.9 and 1.3 for the History and Clinical Examination Part and the Clinical Reasoning Part respectively. The p value was statistically significant for History & Clinical Examination and Clinical Reasoning (p = 0.009 and p = 0.03) respectively indicating that practicing physical therapists performed significantly better than physical therapy students.

### 3.2 Structured policy and standards review

For the reporting of the physical therapy licensing components and policies, 178 reports were identified (websites: 90; organisations: 87; citation searching:7). 7 reports were not possible to retrieve and 4 were excluded because they contained out-of-date information. In total, 166 reports were deemed relevant and included **(Fig 1)**.

#### 3.2.1 Characteristics of the eligible sources of evidence

From the included 166 reports, 87 were official websites of licensure or regulation boards and associations, 3 were websites with specific examination and credential information and 77 were pdf documents retrieved from external links in organisation websites. A total of 17 countries that met the inclusion criteria were identified: Australia, New Zealand, Singapore, Hong Kong, United Kingdom, Ireland, Switzerland, Austria, Netherlands, Sweden, Norway, Denmark, Finland, United Arab Emirates, South Africa, Canada, and United States of America (USA). In Canada, the physical therapy profession is provincially regulated, therefore an additional province-based research approach was used, including 11 of the 13 provinces. Northwest Territories and Nunavut were excluded, because the physical therapy profession is not regulated in these provinces. Similarly, the physical therapy professional regulation is mainly a state responsibility in the USA; therefore, all fifty states of USA were selected. For the United Arab Emirates only, Dubai was used as a reference point. Again, the regulation of the physical therapy profession in United Arab Emirates falls under the responsibility of each district. The rest of the districts were excluded due to lack of reporting on online-available information retrieval.

#### 3.2.2 Education policy

Most of the countries included in the analysis (88%) require a 3–4 year bachelors’ program for entry-level to practice. Canada requires a 2-year Masters level professional program degree following a 3–4 year bachelor degree in a health-related field with specific health course requirements, bringing the total years of study to 5–6 years. The USA requires a 3–4 year doctoral level professional program degree following a bachelor degree, bringing the total years of study to 6 years of education. Only ten out of the 17 countries (59%) report in their program curriculum description a minimum number of supervised clinical hours. Hong Kong is at the lowest end of the range with 800 hours, and Dubai is at the highest end of the range with 2259 hours. Canada reports 1025 hours. The rest of the countries included in the analysis (70%) state that they offer at least 1000 supervised hours, setting the median around 1000 hours. A detailed table with the number of supervised clinical practice hours per country can be seen in **S1 Appendix.**

#### 3.2.3. Examination policy for home graduates

In total, 11 Canadian provinces, Dubai and 50 USA states require additional written entry-level to practice competency exams from their accredited academic program graduates in order for them to become licensed physical therapists. The vast majority (98%) of the provinces / states included in the analysis set the minimum passing score for the written examination at 66%. Dubai requires a score of 55% for the candidates to be successful. Nova Scotia implements an additional Jurisprudence examination on Ethics and Law Regulations for each applicant that is a requirement for registration in the province. Similarly, most of the USA states (62%) require an additional Jurisprudence examination on Ethics and Law. Forty-nine states of the USA (79%) allow a total of 6 attempts to pass the written examination. Florida is the only state that allows 5 attempts. In the Canadian Provinces and Dubai (19%) each graduate is allowed 3 attempts in written exams. Canada is the only country (12%) requiring an extra entry-level to practice OSCE from their home graduates in order for them to become licensed physical therapists. The examination takes place only after successful completion of the written examination and candidates are allowed a total of 3 attempts to pass the examination.

#### 3.2.4. Examination policy for international graduates

Nine of the 17 countries (53%) require international graduates to pass some form of external examination to become fully licensed physical therapists. Eight (47%) countries require graded written examinations, and seven (41%) require an additional graded practical exam. Austria requires overseas qualified physical therapist to pass a non-graded written and practical exam to further examine their skills and knowledge where second attempts are not permitted. The type of exams required by each country can be seen in **Table 3**. The required pass mark also varies across countries: Australia 50%, Dubai 60%, South Africa 60% (combining scores from written and clinical examinations, with a requirement of ≥50% in each component). Canada and the USA both set 66% as the pass mark. The rest of the countries have an exam free policy for international candidates and only implement credentials assessment.

**Table 3:** Examination requirements for international candidates.

COUNTRY/PROVINCE	EXAMS
**Australia**	Written and Clinical
**Singapore**	Written and Clinical
**Hong Kong**	Written and Clinical
**Sweden**	Written and Clinical
**United Arab Emirates (Dubai)**	Written only
**South Africa**	Written and Clinical
**Austria**	Written and Clinical
**Canada (excluding Quebec)**	Written and Clinical
**USA**	Written Only

#### 3.2.5 Pass rates

The latest available published data from the Physiotherapy Competency Exam (PCE) in Canada is for the years 2013–2017. First-time home-educated candidates had a higher percentage of passing rates during these years compared to international-educated graduates. The pass rate for the written examination was 94%, and the pass rate for the clinical examination was 90%. The mean pass rate for first-time international candidates was 78% for the written and 72% for the clinical examination. A detailed per-country report of the pass rates can be seen in **S1 Appendix**. The pass rate of the USA’s National Physical Therapy examination (NPTE) for years 2016–2020 was 92% for USA-educated candidates and 37% for foreign-educated candidates. A detailed per-year report can be seen in **S1 Appendix.**

#### 3.2.6 Exam retake policy for international candidates

Australia and Singapore (25%) offer 1 retake for international graduates. South Africa, Canada and Dubai (37.5%) allow 3 retakes. The rest of the countries (37.5%) allow for more than 3 retakes. The number of opportunities given in Sweden for the theoretical examination is 5. In the USA, each candidate can have 6 attempts to pass the NPTE with an exception of Florida that only allows 5 attempts. The Hong Kong system sets no limit to the number of retakes that a candidate can take. A detailed report of the exam components per country can be seen in **S1 Appendix.**

#### 3.2.7 Mutual agreements for international candidates

Most countries have processes in place to evaluate the credentials and determine what is needed to become certified. In order to facilitate across border mobility of physical therapy professionals, some countries have mutual agreement policies in place to provide faster, exam free registration to candidates coming from countries whose educational and registration system satisfies their credentials. Canadian provinces have a mutual agreement for an application pathway through endorsement, for physical therapy graduates that are registered in a different Canadian province. This pathway is exam-free, except for Nova Scotia that requires a jurisprudence examination on law and ethics regardless of the registration pathway (home, international or endorsement). Similarly, the USA states provide an exam-free registration pathway for physical therapists that are registered in another USA state, with proof of a clear criminal record. The jurisprudence examination policy is still implemented in the same way that it is for home graduates. The countries of the European Union also have a joint agreement for a non-exam qualification pathway. Australia has an exam-free agreement with New Zealand through the Trans-Tasmanian Agreement and a clinical-component-free examination policy for licensed physical therapists from Canada, Hong Kong (SAR of China), the United Kingdom, Ireland, Singapore and South Africa.

#### 3.2.8 Ongoing competency policies

*3*.*2*.*8*.*1 Audit*. From the 76 regulatory bodies included, 69 (90.8%) reported that they perform regular audit controls of random samples of registered physical therapists, inspecting portfolios, continuing professional development hours and patient records. A requirement for annual or biennial declaration of compliance and random audits of records is the standard procedure. The regions where regulatory bodies do not currently report to perform regular audits are Singapore, Hong Kong, Norway, Finland, Manitoba and Massachusetts.

*3*.*2*.*8*.*2 Continuing professional development (CPD)*. Sixty-six (86%) of the included regulatory bodies implement a CPD policy based on a minimum number of hours or credits of involvement. The mean CPD hours per year is 23, with United Kingdom, New Zealand, Ireland and South Africa in the high end with 35, 33, 30 and 30 CPD hours respectively. Dubai, Austria and the USA are in the low end of minimum CPD hours with 10, 12 and 15 hours per year respectively. Canada is slightly below average, requiring 19 CPD hour per year. A report of each country’s yearly CPD hours (including detailed reporting of Canada’s provinces) required for renewal of license is presented in S1 Appendix. For Canada and the USA, the province/state mean number of hours was used for the analysis. Manitoba and Quebec were excluded from the Canadian mean estimate as they do not report a minimum CPD hour policy. Similarly, Maine and Massachusetts were excluded from the USA mean estimate as they do not report a minimum CPD hour policy. Hong Kong, Singapore, Netherlands, Sweden, Norway, Denmark and Finland report to encourage and organise CPD activities on a voluntary base.

*3*.*2*.*8*.*3 Renewal examination*. Only nine (11.8%) of the 76 included regulatory bodies required an examination for renewal. When in place, the examination was a jurisprudence exam, testing the physical therapist’s knowledge on the laws and ethics regarding clinical practice in that jurisdiction. One province from Canada (9.1%) and 8 states from the USA (16%) report an examination-for-renewal policy. The Canadian province is Nova Scotia, and the USA states are Arkansas, Kentucky, Louisiana, Minnesota, New Hampshire, New Jersey, Ohio and Texas.

## 4. Discussion

### 4.1 Principal findings

This study found sparse evidence on PT OSCE measurement properties, and that globally, PT professional programs have a range of program structures, standards and examination processes for facilitating and monitoring entry-level-to practice competency and licensure. The main highlight from the structured review analysis is that Canada is the only country that requires an external clinical competency examination for licensure from domestic physical therapy graduates of an accredited program.

### 4.2 Measurement properties

The pooled estimate of internal consistency (Cronbach’s alpha) was below the acceptable range (0.55, 95% CI: 0.41, 0.67) and is neither in concordance with the average alpha value of 0.67 currently reported by CAPR or with accepted standards for test construction. Some variation in performance between stations is expected in that comprehensive OSCEs are designed to test a wide range of practice skills. However, when these values do not meet established standards, it is legitimate to consider that they may not be measuring accurately. Validation studies were few and mostly correlated OSCEs scores to written test scores; and the few that assessed clinical evaluations during training suggested a weak relationship. OSCE scores correlated well with written exam scores, which might support validity, but also suggest they provide similar information to the written test. No studies addressed whether OSCEs identified future practice problems like patient complaints, or whether they provided additional useful information beyond written tests.

There are several factors that influenced our psychometric findings. The first relates to variations in how the Internal Consistency was calculated. First, it is unclear how the CAPR average alpha value was derived and whether it was properly weighted. Second, the findings of the identified studies are based mainly on an 8-station OSCE while the CAPR average alpha value is calculated based on a 16-station OSCE. Although adding more stations (i.e., CAPR 16-station OSCE) may inflate the value of alpha, this may lead to insufficiency. CAPR does not provide adequate information for each station and if some of the additional 8 stations measure the same outcome this can lead to redundancies and insufficiency (poor alpha).

Another emerging issue is how to define the cut-off point for the acceptable level of internal consistency agreement in a high-stake assessment like the PCE that awards a professional license. One might argue that stations should assess different skills and that a high coefficient might not be needed. However, low correlations might suggest that an underlying latent construct of competency is not being measured. The 0.67 Cronbach’s alpha score to deemed as acceptable on tests that are used to grant licensure to home educated physical therapy professionals is controversial. A recent study that developed and tested the reliability of a physical therapy student performance assessment tool with an OSCE based clinical examination, considered Cronbach’s alpha >0.8 an acceptable level for moderate-stake assessments, such as a final term grade [9]. However, even though implemented in physical therapy students, that study evaluated a tool that was created for assessing student performance of patient education. OSCEs for licensure grant test a much broader field of knowledge and skills and the stakes are much higher. Thus, issues around ratings, regarding what is adequate internal consistency may vary.

The correlations between clinical performance and OSCEs were not strong. Although there is no gold standard comparison, it is a concern that ratings of clinical performance by clinical supervisors taking place over several weeks do not correlate with an OSCE score from the same training period. It might be argued that the clinical observations performed by academic and clinical faculty are more reliable as they are performed based on multiple observations across different patients and contexts. This might partially explain why the correlations between clinical performance and OSCEs were not high. Pearson’s correlations between different station scores were also quite low which means that students that perform well on one station do not necessary perform well on other stations. Although the main reason for this seems to be the difficulty of each station, a recent study stated that the order of the stations could also affect student performance [24]. Our pooled estimate for Pearson correlations between individual station score and total station score indicates that students that perform well in one station have moderate to fairly strong chances of getting a higher total exam score than students that performed poorly in the same station.

The studies that were included in our pooled estimate of the interrater ICC analysis used different kinds of assessors, either by having one of the assessors acting as the SP or by taking a video of the exam session and asking an external examiner to grade the student independently. Our pooled estimate for ICC between assessors falls into the substantial range, which could indicate that the nature of the assessor does not strongly relate to the actual exam score. In terms of the SP, although the pooled estimates for the kappa Coefficient indicate a substantial and an almost perfect level of agreement for intra-rater and inter-rater reliability respectively, the study that provided the data only used one SP that performed at a single station. Thus, the results cannot be generalised for studies that use SP that perform across multiple stations.

In terms of the validity analysis, the correlation values range from very weak to strong relationship. This can be attributed to the different nature of the comparing measurements. The correlation with clinical performance and total GPA grade seems to present the weakest values, while the correlation with an individual course’s grade the strongest values. These findings support that a single performance assessment by an independent observer in an OSCE resemble more a rather narrow, highly specific course examination rather than the broader knowledge and skills that are necessary for clinical practice. These findings also correlate with the ones of relative studies examining students’ and faculty’s perceptions during simulated and real life OSCEs. Although both students and faculty agreed to the use of OSCEs as an effective and innovative educational tool that helps students to develop confidence over a single subject and stimulates the learning process, faculty members reported that OSCEs failed to accurately replicate real life scenarios something that makes them less effective as a clinical licensure examination assessment tool [25–27]. Adding on the negative side, these studies also reported that as a process OSCEs were quite stressful, exhausting both to deliver and to participate in and lengthy [25–27].

It is noteworthy that Canada is the only country that requires a clinical competency examination for licensure from domestic physical therapy graduates of an accredited program. This might be seen as positive if it provides an extra layer of public protection or as negative if it imposes a burden on graduates without providing additional protection of the public. All other countries evaluated used either a written examination or no examination in all for licensure of their home accredited physical therapy programs. We found that Canadian graduates perform better than international ones on both written and practical licensure examinations [28], which might suggest that the examinations have more value for use in evaluating foreign trained therapists who wish to practice in Canada. In the present study, we were not able to investigate possible causal factors due to the restricted sample size and lack of online-available information. However, high pass rates of Canadian program graduates are to be expected since the program’s design their curriculum to meet the needs of their population, and the licensure exams are templated based on Canadian practice analyses. It was not the purpose of this study to determine what factors contribute to international test scores. However, our data illustrate that there are substantial training differences within countries where the profession is well developed. Since many international PTs come to Canada as part of emigration from countries with less developed regulation systems, greater training variation and examination outcomes could be expected.

Overall, there is a major research gap regarding studies analysing data from the actual external licensure examination in Canada, particularly from the perspective of how they differentiate competent and incompetent practitioners. Also, there are no studies that indicate that the externally administered post-professional training OSCE adds value to protection of the public over a national written exam. We conclude that there is insufficient evidence to support or refute the use of OSCEs as a necessary standard for professional licensure in Canada. However, that no other country examined requires a post-professional training OSCE, suggest this requirement may be excessive. Therefore, the OSCE may be an unnecessary burden to trainees in terms of costs and potential consequences of random error in passing; and may restrict access to care for the public -particularly during the pandemic when access to in-person OSCE testing is restricted. However, this undue burden was not quantified in this study and remains uncertain. There is limited supporting evidence of the clinical measurement properties of OSCEs used during professional training, although this evidence is weak and would benefit from further research. A combination of OSCEs during academic training from institutions accredited in Canada and careful monitoring of clinical performance in placements from Canadian clinical sites/preceptors using validated clinical competency tools should be sufficient to identify and ameliorate gaps in clinical skills and reasoning prior to entry -level -to practice. A role for OSCE examinations seems more meritorious for internationally trained PTs where assessment according to Canadian standards and context is needed; and pass rates are lower. Externally validated written national examinations, may be a useful cross check to identify marginal candidates which may have been misclassified by prior academic/clinical evaluations during professional training. However, given the lack of internal consistency and high pass rates, it is also quite possible that random error is contributing to failures. Test anxiety may also be a contributing factor since it has been shown to affect scores. When considering that countries with a well-developed regulation system have multiple processes in place to evaluate ongoing competency of PTs the rationale for an immediate post training OSCE ensuring licensure is less likely to provide real benefit to public protection. The current practice in Canada of using an OSCE that demonstrates below acceptable internal consistency and validity limits justification of its use, as good results may not necessarily translate into good performance in the clinical environment. Therefore, OSCEs may not accurately reflect the competence of individuals.

### 4.3 Limitations

Our review of measurement properties was limited by the low volume and quality of research examining OSCEs so that our pooled analysis was based on only 6 studies that have been conducted in the last 20 years. The benchmarks that we used for reliability and validity measurements derive from behavioural studies and studies that examining patient education performance, so they should be addressed with caution. In our structured policies and standards review we included 17 countries where the physical therapy profession was regulated and well-developed. Thus our data does not apply to countries where the profession is less well developed. However, since our findings indicated better test outcomes for Canadian graduates that internationally trained therapists, these differences would likely only be further accentuated in therapist coming from less-developed countries. Caution should be taken when generalising our findings in countries not included in the present study. Currently only Canada (excluding Quebec) implements a clinical examination for home graduates in comparison to 16 other countries which means that the evidence relates to in-training OSCEs, not post-professional training OSCEs. The logic for using OSCE during professional training is sound, but may not extend to post-professional licensure. Since an OSCE requires potentially large distance travel in Canada and large administrative costs ($2732) for students after completing accredited professional programs, the OSCE should only be implemented as a requirement for licensure if it can provide value add to the systems in place for evaluating competency during training. That is adds value to the public protection in addition to that provided by the accreditation of professional programs, national written licensure examination, and ongoing college monitoring systems. At present the evidence is insufficient to suggest that value exists.

### 4.4 Implications

Our review suggests that licensure OSCEs for national professional program graduates have not been justified by formal research studies or by international practice standards. From a policy perspective this means that licensure OCES for national graduates could be discontinued to reduce the burden on new graduates entering the profession while maintaining public protection. However, while the usefulness of the OSCE appears limited from the current results, the undue burden is not as certain and should be evaluated. Furthermore, the value for international graduates requires further investigation. The evidence is more supportive of the use of OSCEs during professional training and yet the evidence has many gaps. Further research on their measurement properties, how they are best constructed, how they should be distributed across the curriculum, optimal methods of scoring and interpretation, their uses as formative and summative assessment, rater effects, and relationships to performance in clinical settings are all avenues of needed investigation. The overall issue of which graduates of professional programs might pose a future risk to the public is important and warrants research that considers the issue more holistically evaluating training, personal factors and contextual factors that contribute to different types of adverse public protection outcomes e.g., physical/mental abuse, billing misconduct, poor clinical decision-making, inadequate skill in providing interventions and ethics breaches. This is a separate body of literature that was not reviewed in this study.

## 5. Conclusions

Canada (excluding Quebec) is the only country out of 17 implementing a clinical competency examination to their home physical therapy graduates for licensure. Based on the evidence of measurement properties of OSCE used during clinical training, a lack of studies for OSCE reliability or validity used during licensure, and the fact that this standard is not required in other countries with well-developed physiotherapy regulation systems, we concluded that an immediate post-professional OSCE as a requirement for licensure of Canadian program graduates is unnecessary, while the undue burden on healthcare professionals remains uncertain.

## Supporting information

S1 Checklist PRISMA 2020 checklist. (PDF)

S1 Appendix Web appendix. (DOCX)

S1 Dataset (DOCX)
