# Supplementary material for: A systematic review and meta-analysis of measurement properties of objective structured clinical examinations used in physical therapy licensure and a structured review of licensure practices in countries with well-developed regulation systems
Source: PLoS One. 2021 Aug 3;16(8):e0255696. doi: 10.1371/journal.pone.0255696 (PMC8330929; doi:10.1371/journal.pone.0255696)
Supplement: S1 Appendix — (DOCX) [file pone.0255696.s002.docx]

**S1 Appendix. Web Appendix**

A systematic review and meta-analysis of measurement properties of objective structured clinical examinations used in physical therapy licensure and a structured review of licensure practices in developed countries.

Contents

**[S1 Web appendix. Study protocol](#_Toc76475010)** [2](#_Toc76475010)

[**S2 Web appendix. Research Strategy** 12](#_Toc76475011)

[**S3 Web appendix. Number of physical therapists per 10 000 population** 14](#_Toc76475012)

[**S4 Web appendix. Sensitivity analysis Cronbach’s alpha** 15](#_Toc76475013)

[**S5 Web appendix. Sensitivity analysis of Intraclass Correlation Coefficient** 16](#_Toc76475014)

[**S6 Web appendix.** **The number of supervised clinical practice hours per country** 19](#_Toc76475015)

[**S7 Web appendix. Canadian Physiotherapy Competency Exam passing rates for first-time international candidates 2013-2017** 20](#_Toc76475016)

[**S8 Web appendix**. **NPTE Passing Rates 2016-2020** 21](#_Toc76475017)

[**S9 Web appendix. Extensive exam components and timings per country** 22](#_Toc76475018)

[**S10. Web appendix. Yearly CPD hours required for renewal of license (adjusted)** 26](#_Toc76475019)

[**S11 Web appendix. Dataset** 28](#_Toc76475020)

# **Web appendix 1. Study protocol**

**PROSPERO**

**International prospective register of systematic reviews**


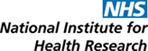


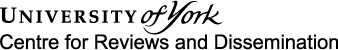


Systematic review

* Review title.

Give the title of the review in English

Validity and reliability evidence of objective structured clinical examinations and a critical analysis of physical therapy licensing components and policies in highly developed countries. A systematic review with meta- analysis

Original language title.

For reviews in languages other than English, give the title in the original language. This will be displayed with the English language title.

* Anticipated or actual start date.

Give the date the systematic review started or is expected to start. 01/04/2021

* Anticipated completion date.

Give the date by which the review is expected to be completed. 31/08/2021

* Stage of review at time of this submission.

Tick the boxes to show which review tasks have been started and which have been completed. Update this field each time any amendments are made to a published record.

**Reviews that have started data extraction (at the time of initial submission) are not eligible for inclusion in PROSPERO**. If there is later evidence that incorrect status and/or completion date has been supplied, the published PROSPERO record will be marked as retracted.

This field uses answers to initial screening questions. It cannot be edited until after registration. The review has not yet started: No

**Review stage Started Completed**

Preliminary searches Yes No

Piloting of the study selection process Yes No

Formal screening of search results against eligibility criteria No No

Data extraction No No

Risk of bias (quality) assessment No No

Data analysis No No

Provide any other relevant information about the stage of the review here.

* Named contact.

The named contact is the guarantor for the accuracy of the information in the register record. This may be any member of the review team.

Pavlos Bobos

Email salutation (e.g. "Dr Smith" or "Joanne") for correspondence:

Dr Bobos

* Named contact email.

Give the electronic email address of the named contact. [pbobos@uwo.ca](mailto:pbobos@uwo.ca)

Named contact address

Give the full institutional/organisational postal address for the named contact.

1151 Richmond Street

Named contact phone number.

Give the telephone number for the named contact, including international dialling code.

+1-519-870-5145

* Organisational affiliation of the review.

Full title of the organisational affiliations for this review and website address if available. This field may be completed as 'None' if the review is not affiliated to any organisation.

Western University

Organisation web address:

https://uwo.ca/

* Review team members and their organisational affiliations.

Give the personal details and the organisational affiliations of each member of the review team. Affiliation refers to groups or organisations to which review team members belong. **NOTE: email and country now MUST be entered for each person, unless you are amending a published record.**

Dr Pavlos Bobos. Western University

Dimitra Pouliopoulou. National and Kapodistrian University of Athens Dr Alexandra Harris. Western University

Assistant/Associate Professor Jackie Sadi. Western University Professor Alison Rushton. Western University

Professor Joy MacDermid. Western University

* Funding sources/sponsors.

Details of the individuals, organizations, groups, companies or other legal entities who have funded or sponsored the review.

The authors did not receive any funding to conduct this study.

Grant number(s)

State the funder, grant or award number and the date of award

* Conflicts of interest.

List actual or perceived conflicts of interest (financial or academic). None

Collaborators.

Give the name and affiliation of any individuals or organisations who are working on the review but who are not listed as review team members. **NOTE: email and country must be completed for each person, unless you are amending a published record.**

* Review question.

State the review question(s) clearly and precisely. It may be appropriate to break very broad questions down into a series of related more specific questions. Questions may be framed or refined using PI(E)COS or similar where relevant.

The first aim of this study will be to assess the internal validity and reliability of the Objective Structured Clinical Examination (OSCEs) as a clinical competency assessment tool for physical therapy graduates. The second aim of this study will be to report the physical therapy licensing components and policies in seventeen countries around the world.

* Searches.

State the sources that will be searched (e.g. Medline). Give the search dates, and any restrictions (e.g. language or publication date). Do NOT enter the full search strategy (it may be provided as a link or attachment below.)

An electronic search will be performed in four databases (PubMed, EMBASE, Google Scholar and CINAHL) from inception to May 2021. A combination of search terms will be used such as “Clinical competence”, “Clinical performance”, “Workplace performance”,” “Summative assessment”, “Objective Structured

Clinical Examination”, “OSCE”, “Reliability”, “Validity”, “Physiotherapy or Physical therapy”. A grey literature search will also be conducted through the Google web search engine. References will also be manually searched from retrieved articles.

URL to search strategy.

Upload a file with your search strategy, or an example of a search strategy for a specific database, (including the keywords) in pdf or word format. In doing so you are consenting to the file being made publicly accessible. Or provide a URL or link to the strategy. Do NOT provide links to your search **results**.

Alternatively, upload your search strategy to CRD in pdf format. Please note that by doing so you are consenting to the file being made publicly accessible.

Do not make this file publicly available until the review is complete

* Condition or domain being studied.

Give a short description of the disease, condition or healthcare domain being studied in your systematic review.

The main clinical component that will be studied is the reliability and validity of Objective Structured Clinical Examinations (OSCEs) as a licensure competency assessment. OSCEs are an interactive form of examination that are highly structured, incorporating case scenarios, uniform grading schemes, and standardized patients who interact with the students during the exam to provide a comprehensive evaluation of each student. The competencies tested on the examination are evaluated through a timed examination, with individual station times that range between five to ten minutes and with multiple stations varying from 5 to 40 stations.

* Participants/population.

Specify the participants or populations being studied in the review. The preferred format includes details of both inclusion and exclusion criteria.

Physical therapy graduates/students/residents/interns

* Intervention(s), exposure(s).

Give full and clear descriptions or definitions of the interventions or the exposures to be reviewed. The preferred format includes details of both inclusion and exclusion criteria.

Not applicable

* Comparator(s)/control.

Where relevant, give details of the alternatives against which the intervention/exposure will be compared (e.g. another intervention or a non-exposed control group). The preferred format includes details of both inclusion and exclusion criteria.

Not applicable

* Types of study to be included.

Give details of the study designs (e.g. RCT) that are eligible for inclusion in the review. The preferred format includes both inclusion and exclusion criteria. If there are no restrictions on the types of study, this should be stated.

For the assessment of the internal validity and reliability of the Objective Structured Clinical Examination (OSCEs) as a clinical competency assessment tool for physical therapy graduates, we will include peer- reviewed articles and pre-prints that will report reliability and validity statistics for OSCEs in the physiotherapy profession without posing any language restriction. The additional information for the licensing components and policies from the different countries will be mainly derived from websites of professional regulatory bodies and associations. These websites will be searched throughout for information regarding education and license requirements and quality assurance methods. To gather as much information as possible, apart from the initial webpage, up-to-date external links on program reports, presentations, law and regulation documents, applicant instructions documents, and application forms will also be scanned. We will exclude peer reviewed articles that do not report reliability or validity statistics for OSCEs and studies that examine other medical professions

Context.

Give summary details of the setting or other relevant characteristics, which help define the inclusion or exclusion criteria.

The additional information for the licensing components and policies from the different countries will mainly derive from websites of professional regulatory bodies and associations. These websites will be searched throughout for information regarding education and license requirements and quality assurance methods. To gather as much information as possible, apart from the initial webpage, up-to-date external links on program reports, presentations, law and regulation documents, applicant instructions documents and application forms the will be also scanned. In order to generate a broad picture of the types of education and professional quality assurance systems implemented on a worldwide scale, a selective global scan of seventeen countries will be done. The selection will be primary based on best-available, systematically recorded, online accessible data and if they present the following criteria: 1) physical therapy profession being long established and 2) the PT profession is regulated and with a large member base (10, 000 population).

* Main outcome(s).

Give the pre-specified main (most important) outcomes of the review, including details of how the outcome is defined and measured and when these measurement are made, if these are part of the review inclusion criteria.

The reliability (e.g. Cronbach's alpha) and validity (e.g. concurrent validity) of OSCE's will be the primary outcomes of interests. Cronbach's alpha coefficient vary between 0 and 1 with low scores indicating poor reliability and high scores excellent reliability. The physical therapy licensing components and policies regarding minimum years of education, minimum supervised clinical hours in the curriculum, competency examination and licensure policy for domestic and international candidates, passing scores, passing rates, retakes policy, jurisprudence examination policy, license renewal policy, number of years to renewal,

minimum continuing professional development hours and audit will be reported, for countries around the world.

Measures of effect

Please specify the effect measure(s) for you main outcome(s) e.g. relative risks, odds ratios, risk difference, and/or 'number needed to treat.

Pooled estimates of reliability or validity statistics such as: Cronbach's alpha, intra-class correlation coefficient (ICC), pearson correlation coefficient or spearman's correlation coefficients

* Additional outcome(s).

List the pre-specified additional outcomes of the review, with a similar level of detail to that required for main outcomes. Where there are no additional outcomes please state ‘None’ or ‘Not applicable’ as appropriate

to the review not applicable

Measures of effect

Please specify the effect measure(s) for you additional outcome(s) e.g. relative risks, odds ratios, risk difference, and/or 'number needed to treat.

Pooled estimates on reliability or validity coefficients.

* Data extraction (selection and coding).

Describe how studies will be selected for inclusion. State what data will be extracted or obtained. State how this will be done and recorded.

Two authors will independently extract and import in duplicate for all the peer-reviewed and grey literature data into a web database (Covidence). The data extraction form will be calibrated between two authors. For the peer-reviewed and pre-print articles, we extracted information on author, year, country, OSCE, time duration, number of OSCE stations, number of examiners, sample size, and reliability and validity statistics. Information that will be extracted for the licensing components and policies in the different countries included minimum years of education, minimum supervised clinical hours in the curriculum, competency examination and licensure policy for domestic and international candidates, passing scores, passing rates, retakes policy, jurisprudence examination policy, license renewal policy, number of years to renewal, minimum continuing professional development hours and audit policy.

* Risk of bias (quality) assessment.

State which characteristics of the studies will be assessed and/or any formal risk of bias/quality assessment tools that will be used.

A summary score for the overall quality of individual studies will be appraised with the use of a structured clinical measurement-specific appraisal tool. The evaluation criteria of this tool will include twelve items: 1) Thorough literature review to define the research question; 2) Specific inclusion/exclusion criteria; 3) Specific hypotheses; 4) Appropriate scope of psychometric properties; 5) Sample size; 6) Follow-up; 7) The authors techniques were standardized; 9) Data were presented for each hypothesis; 10) Appropriate statistics-point estimates; 11) Appropriate statistical error estimates, and 12) Valid conclusions and recommendations. An article’s total score – quality - will be calculated by the sum of scores for each item, divided by the numbers of items, and multiplied by 100%. Overall, the quality summary of appraised articles will range from

(0%-30%) Poor, (31%-50%) Fair, (51%-70%) Good, (71%-90%) Very Good, and (90%) Excellent.

* Strategy for data synthesis.

Describe the methods you plan to use to synthesise data. This **must not be generic text** but should be **specific to your review** and describe how the proposed approach will be applied to your data. If meta- analysis is planned, describe the models to be used, methods to explore statistical heterogeneity, and software package to be used.

A meta-analysis of reliability or validity coefficients will be performed using STATA (StataCorp. 2019. Stata Statistical Software: Release 16. College Station, TX: StataCorp LLC) with a “meta” package. We will deploy a random-effects maximum likelihood model. The extracted coefficients will be converted to z values.

Heterogeneity will be deemed substantial if I² values are greater than 50%. Forest plots will be created using 95% CIs for coefficient estimates. We will perform univariate meta-regressions if statistical heterogeneity will be deemed substantial (greater than 50%) on pre-specified covariates such as Country and field of OSCE (e.g., musculoskeletal). Publication bias will be evaluated through funnel plots. In the presence of publication bias, we will impute the missing studies to account for publication bias in the meta-analysis. We will compare the observed and the imputed studies vs observed only by using the non-parametric “trim and fill” method.

Data for the licensing components and policies from different countries will be synthesized with descriptive analysis by reporting frequencies and percentages for home graduates and for international applicants.

* Analysis of subgroups or subsets.

State any planned investigation of ‘subgroups’. Be clear and specific about which type of study or participant will be included in each group or covariate investigated. State the planned analytic approach.

Pre-specified covariates for a subgroup analysis will be the type of OSCE station and the Country that the OSCE exam was investigated.

* Type and method of review.

Select the type of review, review method and health area from the lists below.

Type of review Cost effectiveness No

Diagnostic No

Epidemiologic No

Individual patient data (IPD) meta-analysis No

Intervention No

Living systematic review No

Meta-analysis Yes

Methodology No

Narrative synthesis No

Network meta-analysis No

Pre-clinical No

Prevention No

Prognostic No

Prospective meta-analysis (PMA) No

Review of reviews No

Service delivery No

Synthesis of qualitative studies No

Systematic review Yes

Other No

Health area of the review Alcohol/substance misuse/abuse No

Blood and immune system No

Cancer No

Cardiovascular No

Care of the elderly No

Child health No

Complementary therapies No

COVID-19

No

Crime and justice No

Dental No

Digestive system No

Ear, nose and throat No

Education Yes

Endocrine and metabolic disorders No

Eye disorders No

General interest No

Genetics No

Health inequalities/health equity No

Infections and infestations No

International development No

Mental health and behavioural conditions No

Musculoskeletal No

Neurological No

Nursing No

Obstetrics and gynaecology No

Oral health No

Palliative care No

Perioperative care No

Physiotherapy Yes

Pregnancy and childbirth No

Public health (including social determinants of health) No

Rehabilitation No

Respiratory disorders No

Service delivery No

Skin disorders No

Social care No

Surgery No

Tropical Medicine No

Urological No

Wounds, injuries and accidents No

Violence and abuse No

Language.

Select each language individually to add it to the list below, use the bin icon to remove any added in error. English

There is not an English language summary

* Country.

Select the country in which the review is being carried out. For multi-national collaborations select all the countries involved.

Canada

Other registration details.

tute) together with any unique identification number assigned by them. If extracted data will be stored and made available through a repository such as the Systematic Review Data Repository

(SRDR), details and a link should be included here. If none, leave blank.

Reference and/or URL for published protocol.

If the protocol for this review is published provide details (authors, title and journal details, preferably in Vancouver format)

Add web link to the published protocol.

Or, upload your published protocol here in pdf format. Note that the upload will be publicly accessible. No I do not make this file publicly available until the review is complete

Please note that the information required in the PROSPERO registration form must be completed in full even if access to a protocol is given.

Dissemination plans.

Do you intend to publish the review on completion?

Yes

Give brief details of plans for communicating review findings.?

Results will be disseminated through international physiotherapy conferences and the manuscript will be published in an open access peer reviewed journal

Keywords.

Give words or phrases that best describe the review. Separate keywords with a semicolon or new line. Keywords help PROSPERO users find your review (keywords do not appear in the public record but are included in searches). Be as specific and precise as possible. Avoid acronyms and abbreviations unless these are in wide use.

objective structured clinical examinations; OSCEs; reliability; validity; physiotherapy; competency examinations;

Details of any existing review of the same topic by the same authors.

If you are registering an update of an existing review give details of the earlier versions and include a full bibliographic reference, if available.

* Current review status.

Update review status when the review is completed and when it is published.New registrations must be ongoing so this field is not editable for initial submission.

Please provide anticipated publication date Review_Ongoing

Any additional information.

Provide any other information relevant to the registration of this review.

Details of final report/publication(s) or preprints if available.

Leave empty until publication details are available OR you have a link to a preprint (NOTE: this field is not editable for initial submission). List authors, title and journal details preferably in Vancouver format.

# **Web appendix 2. Research Strategy**

MEDLINE-OVID

1. exp "physiotherapy and process assessment (health care)"/ or "outcome assessment (physical therapy)"/ or Clinical competence/

2. objective structured clinical examinations?.ti.

3. exp " objective structured clinical examinations"/

4. Competency Measurement/

5. exp physiotherapy evaluation/

6. "Rehabilitation"/

7. Examinations/

8. OSCE.tw.

9. ((reliability or validity) adj2 (measure? or scale? or evaluation?)).tw.

10. Workplace performance.tw.

11. (Competency adj2 (measure? or scale? or evaluation?)).tw.

12. (Physiotherapy adj2 (measure* or scale? or indicator?)).tw.

13. or/1-12

14. "Summative assessment"/

15. exp "Sensitivity and Specificity"/

16. reliability.mp.

17. validity.mp.

18. responsiveness.mp.

19. Psychometrics/

20. rasch.mp.

21. factor analysis, statistical/

22. factor analysis.tw.

23. differential functioning.mp.

24. (validity or validation).mp. [mp=title, original title, abstract, name of substance word, subject heading word, unique identifier]

25. (validity or validation).mp.

26. item difficulty.mp.

27. guidelines as topic/

28. practice guidelines as topic/

29. guideline.pt.

30. practice guideline.pt.

31. (guideline? or guidance or recommendations).ti.

32. consensus.ti.

33. or/27-33

34. meta-analysis/

35. exp meta-analysis as topic/

36. (meta analy* or metaanaly* or met analy* or metanaly*).tw.

37. (collaborative research or collaborative review* or collaborative overview*).tw.

38. (integrative research or integrative review* or intergrative overview*).tw.

39. (quantitative adj3 (research or review* or overview*)).tw.

40. (research integration or research overview*).tw.

41. (systematic* adj3 (review* or overview*)).tw.

42. (methodologic* adj3 (review* or overview*)).tw.

43. exp technology assessment medical/

44. (hta or thas or technology exam assessment*).tw.

45. ((hand adj2 search*) or (manual* adj search*)).tw.

46. ((electronic adj database*) or (bibliographic* adj database*)).tw.

47. ((data adj2 abstract*) or (data adj2 extract*)).tw.

48. (analys* adj3 (pool or pooled or pooling)).tw.

49. mantel haenszel.tw.

50. (cohrane or pubmed or pub med or medline or embase or psycinfo or psyclit or psychinfo or psychlit or cinahl or science citation indes).ab

# **Web appendix 3. Number of physical therapists per 10 000 population**

| Country/province | # Physio Practitioners/ 10 000 |
| --- | --- |
| Australia | 14.25 |
| New Zealand | 11.67 |
| Singapore | 3.58 |
| Hong Kong | 4.75 |
| United Kingdom | 8.3 |
| Ireland | 10.51 |
| Switzerland | 21.14 |
| Austria | 17.61 |
| Netherlands | 20.77 |
| Sweden | 16.79 |
| Norway | 24.88 |
| Denmark | 26 |
| Finland | 21.75 |
| United Arab Emirates (Dubai) | 2.98 |
| South Africa | 1.37 |
| Canada | 6.83 |
| USA | 6.84 |

# **Web appendix 4. Sensitivity analysis Cronbach’s alpha**

Sensitivity analysis with Swift 2007 study of Cronbach’s alpha on OSCEs in physiotherapy students. Forest plot presenting the meta-analysed estimate on Cronbach’s alpha. Each square presents the results of an individual study with the size of the square being proportional to the weights used in the meta-analysis and the horizontal lines indicating the 95% confidence intervals. The solid vertical line represents no reliability, and the solid diamond indicates the overall summary measure.

# **Web appendix 5. Sensitivity analysis of Intraclass Correlation Coefficient**

Sensitivity analysis including Swift 2007 of Intraclass Correlation Coefficient (ICC) on OSCEs in physiotherapy students. Forest plot presenting the meta-analyzed estimate on ICC. Each square presents the results of an individual study with the size of the square being proportional to the weights used in the meta-analysis and the horizontal lines indicating the 95% confidence intervals. The solid diamond indicates the overall summary measure.

# **Web appendix 6.** **The number of supervised clinical practice hours per country**

| Country/province | Minimum Clinical Hours in Curriculum |
| --- | --- |
| Australia | 1000h (Newcastle University) |
| New Zealand | 1000h |
| Singapore | 1000h (Singapore Institute of Technology) |
| Hong Kong | 800h |
| United Kingdom | 1000h |
| Ireland | 1000h |
| United Arab Emirates (Dubai) | 2259h (Gulf Medical University) |
| South Africa | 1000h |
| Canada | 1000h |
| USA | 1000h |

# **Web appendix 7. Canadian Physiotherapy Competency Exam passing rates for first-time international candidates 2013-2017**

| Country | Written Exam Passing Rate | Clinical Exam Passing Rate |
| --- | --- | --- |
| USA | 84% | 61% |
| Australia | 77% | 76% |
| New Zealand | 81% | 69% |
| UK | 62% | 72% |
| Ireland | 73% | 85% |
| South Africa | 89% | 67% |
| Netherlands | 47% | 36% |

# **Web appendix 8**. **NPTE Passing Rates 2016-2020**

|  | 2020 | 2019 | 2018 | 2017 | 2016 |
| --- | --- | --- | --- | --- | --- |
| Graduates of US Accredited PT Programs | 91% | 91% | 91% | 93% | 93% |
| Graduates of Non-US PT Programs | 42% | 34% | 27% | 39% | 44% |
| First-Time Candidates - All | 89% | 86% | 86% | 87% | 87% |

# **Web appendix 9. Extensive exam components and timings per country**

Australia’s Written Assessment assesses the knowledge, problem-solving and decision-making skills required for safe and competent practice of physiotherapy in Australia. The assessment comprises two papers, each with fifteen cases. Each case has four questions attached. Each paper is two hours in duration - one conducted in the morning and one in the afternoon of the same day. The assessment is conducted online and is held 4 times a year, in March, June, September, and December. All four sessions are available to be completed by remote invigilation or in-person. To assist candidates in familiarising themselves with the online format of the exam, a free practice test is provided to registered candidates. Written Assessment results are e-mailed to candidates within 6 weeks from the day of the exam. Each candidate gets 6 months after completion of the written examination to pass the Clinical one. The Clinical Assessment usually takes around seventy-five minutes. It consists of three practical assessments, one each in musculoskeletal, neurological, and cardiorespiratory physiotherapy. All three assessments are conducted in a simulated environment with standardised patients at the Council’s Simulation Lab in Melbourne. To pass the Clinical Assessment, each candidate must demonstrate the ability to independently practice and undertake a safe and effective consultation in all three areas. The candidate’s performance is observed and assessed by a panel of two Council appointed assessors, one Specialist and one Generalist.

In Singapore, both parts of the examination take place three times per year, in January, April and August and there is an application deadline 8 weeks before the examination. The examination is administered by the Singapore Institute of Technology and the National University of Singapore on behalf of the Allied Health Professionals Council. All examinations are conducted in-person. The assessment tests the equivalency of the professional knowledge and skills expected of practitioners in the regulated professions in Singapore. Results are released four to six weeks after the examination, and no appeals are allowed.

Dubai sets an online-based examination that is composed of 150 multiple choice questions. The duration of the examination is three hours and forty-five minutes. There are eleven exam content areas, each one taking up a different percentage of the final grade. These are Kinesiology, Biomechanics and Ergonomics (8%), Anatomy and Physiology (12%), Clinical Decision Making and Differential Diagnosis (8%), Physical agents (10%), Therapeutic Exercises and Techniques (10%), Physical Therapy in Special Population (OBGYN, Paediatrics, Geriatrics, 12%), Material Management (10%), Cardiopulmonary Physical Therapy (8%), Sports Physical Therapy (8%), Neurorehabilitation (12%) and Musculoskeletal rehabilitation 12%. The result is available immediately once the exam is completed.

South Africa sets a minimum threshold of 60% for candidates to successfully complete the examination, requiring a score of at least 50% on each individual component. The examination is conducted during April / May and September / October annually, depending on the number of candidates. The purpose of the examination is to evaluate the candidate’s theoretical and clinical/practical knowledge and competence in physiotherapy, test the candidate’s knowledge of the laws, ethical rules and regulations relating to the practising of physiotherapy in South Africa and satisfy the Professional Board of proficiency in English. The standard expected is measured against the minimum standards expected of practitioners qualified in South Africa. The examination is composed of two parts, a theoretical and a clinical examination. The theoretical examination lasts three hours. This paper is composed of two sections. The first section relates to the ethical rules and regulations under which a practitioner may practise his/her profession in South Africa. The second section examines the practice of the profession. Background knowledge of the practice of the profession and application of physiotherapy techniques and modalities will be tested in this section. All aspects of pathology, anatomy, physiology and clinical sciences including internal medicine, cardio-pulmonary, surgery, orthopaedics, neurology, gynaecology and obstetrics, community health, psychiatry, paediatrics, geriatrics and other relevant fields may be tested in this section. Particular emphasis is placed on the fields of cardio-pulmonary, intensive care, neurology and orthopaedics in the examination. At the clinical examination candidates are required to evaluate one patient and treat another patient. The patients may be either acute, chronic or at a rehabilitation stage of treatment. The main categories of patients selected for examination are orthopaedic and rheumatology, traumatology, neurology, cardio-pulmonary including both medical and surgical patients, Critical Care, paediatrics and outpatients. The time allowed for each of the components of the clinical examination (evaluation and treatment) is 1 hour. An abbreviated evaluation form is to be completed and handed in before the treatment of the patient. The candidates are graded by one examiner and one moderator.

Lund University in Sweden has been commissioned by the National Board of Health and Welfare to be nationally responsible for and carry out knowledge tests for physiotherapists trained overseas. The knowledge test shall test the knowledge and skills required to practice the profession in a patient-safe manner in relation to the degree objectives in the Higher Education Ordinance. The test contains a theoretical part and a practical part. Each candidate must pass the theoretical part before he can do the practical part. The theoretical part is conducted three times per year with an application deadline a month prior to the examination. The practical test is conducted twice per year. There is no application process, a summons is sent by Lund University after successful completion of the theoretical examination. The final decision will be sent by email when a candidate has passed successfully both parts of the knowledge test. The theoretical knowledge test measures the knowledge, understanding and judgment required to be able to work as a physiotherapist in Sweden. The test is divided into two parts and both are performed on the same day.

- Sub-test 1: A written examination consisting of multiple-choice questions. The questions address the areas of the musculoskeletal system, neurology (including geriatrics), cardiorespiratory, psychosomatics and psychiatry. The duration of the sub-test 1 is one hour and thirty minutes.
- Sub-sample 2: A patient case on the areas of the musculoskeletal system, neurology (including geriatrics), cardiorespiratory, as well as psychosomatics and psychiatry must be analysed according to established guidelines. The case is sent to the participants three weeks before the theoretical knowledge test and must be sent back one week before the test takes place. The case is presented orally on the test day.

The practical test measures skills, abilities and attitudes that physiotherapists need to possess in order to be able to work in Sweden, such as clinical examination, assessment and suggestions for treatment. The assessments are made on the basis of case descriptions and / or video-recorded cases.

The Physiotherapy Competency Examination (PCE) in Canada tests whether qualified exam candidates have demonstrated a minimum standard of practice, by fairly and accurately evaluating the competencies one needs to have to practise physiotherapy through the assessment of essential knowledge, skills and abilities. It tests history-taking, physical examination, data interpretation, clinical problem solving, treatment techniques, ethics, safety, interviewing and communication. The exam covers core clinical practice areas: such as neuromusculoskeletal, neurological, cardiopulmonary-vascular and multisystem. The PCE has two components. The first is written and the second is clinical. Candidates must pass the Written Component (Qualifying Exam) to be eligible to attempt the Clinical Component (Physiotherapy National Exam). The two parts of the Physiotherapy Competency Examination are graded independently.

The Written Component is a multiple-choice exam that tests the candidate’s understanding of the principles and processes of physiotherapy practice. It consists of 200 multiple choice questions with a total time to complete in four hours. There are 100 questions on the neuromusculoskeletal field taking up a 50% weight of the overall score, 40 neurological-based questions with a 20% weighted percentage, 30 cardiopulmonary - vascular questions with a 15% weighted percentage and 30 multisystem questions taking up the remaining 15%. To pass the Written Component, one must achieve an overall score that meets or exceeds the passing score (on the standard score scale). The Clinical Component tests the candidate’s ability to safely and effectively apply the principles and processes of physiotherapy practice. There are eight five-minute stations and eight ten-minute stations in the Clinical Component, consisting of five and ten-minute encounters respectively. The Clinical Component of the Physiotherapy Competency Examination is graded based on three criteria, how many checklist items one did correctly, how the examiners rated the candidate’s performance and how he scored on the written portion of the station. Each candidate must meet all three criteria in order to pass the exam. Results for the Written Component are be sent by mail to the applicant’s address within 6 weeks of the examination. Results for the Clinical Component are mailed within 12 weeks of the exam. Exam results (Pass/Fail) are also posted online on the same day that they are mailed.

The National Physical Therapy Exams (NPTE) in USA are designed to assess a candidate’s basic entry-level competence after graduation from an accredited program or from an equivalent non-accredited program. The Federation of States Board for Physical Therapy (FSPBT) is responsible to develop, maintain, and administers the NPTE to help ensure that only those individuals who have the requisite knowledge of physical therapy are licensed in the physical therapy field, and to help regulatory authorities evaluate candidates and provide standards that are comparable from jurisdiction to jurisdiction. The examinations consist of 250 objective, multiple-choice questions covering the major areas of physical therapy. The NPTE exams are conducted four times per year. Approximately five business days after the exam, FSBPT will transmit each candidate score to the licensing authority to which he originally applied for licensure. At that time the pass/fail status will also be available online. Although each federal state regulates independently the maximum number of tries, the FSBPT will only allow participants to take the examination a maximum of three times in any twelve-month period.

Australia and New Zealand allow both recent graduates and registered physiotherapists exam free license through the Trans-Tasmanian Agreement. Australia applies an additional agreement policy through FLYR stream. Candidates from Canada, Hong Kong (SAR of China), the United Kingdom, Ireland, Singapore and South Africa can bypass clinical examination and qualify after successful completion of the written part of the exams. The countries of the European Union have a joint agreement for a non-exam qualification pathway. There is currently a transitional period for UK applicants that started the qualification process before Brexit. All post-Brexit applicants have to follow the International Graduates’ Pathway. Canada provinces have a mutual agreement for an application pathway through endorsement, for graduates that registered in a different province. This pathway is exam-free, with an exception of Nova Scotia that requires a jurisprudence examination on law and ethics regardless of the registration pathway (home, international or endorsement). Similarly, the USA federal districts provide an exam-free registration pathway (in terms of the national examination) for physical therapists that are registered in another province, providing they have a clear criminal record. The jurisprudence examination policy is implemented the same way that it does on home graduates.

# **Web appendix 10. Yearly CPD hours required for renewal of license (adjusted)**

| COUNTRY/PROVINCE | CPD hours/year (adjusted) |
| --- | --- |
| Australia | 20 |
| New Zealand | 33.3 |
| United Kingdom | 35 |
| Ireland | 30 |
| Austria | 12 |
| United Arab Emirates (Dubai) | 10 |
| South Africa | 30 |
| Canada | 19.1 |
| Alberta | 20 |
| British Columbia | 20 |
| New Brunswick | 20 |
| Newfoundland and Labrador | 20 |
| Nova Scotia | 20 |
| Ontario | 20 |
| Prince Edwart Island | 20 |
| Saskatchewan | 20 |
| Yukon | 12 |
| USA | 14.6 |
