# Supplementary material for: A systematic review and meta-analysis of measurement properties of objective structured clinical examinations used in physical therapy licensure and a structured review of licensure practices in countries with well-developed regulation systems
Source: PLoS One. 2021 Aug 3;16(8):e0255696. doi: 10.1371/journal.pone.0255696 (PMC8330929; doi:10.1371/journal.pone.0255696)
Supplement: S1 Dataset — (DOCX) [file pone.0255696.s003.docx]

# **S1 Dataset**

| Author (year) | Cronbach's alpha | Intraclass Correlation (ICC) between examiners | Confidence Intervals Between Examiners | Pearson Correlation (r) Between station | Pearson Correlation (r) compared with total score | Interrater reliability of the Simulated Patient (k) | Intrarater reliability of the Simulated Patient (k) | Pearson Correlation (r) GPA | Pearson Correlation (r) Course Grade | Pearson Correlation (r) Traditional Exam (Control) | Spearman Rank correlation (ρ) Clinical Performance | Effect size |
| --- | --- | --- | --- | --- | --- | --- | --- | --- | --- | --- | --- | --- |
| Wessel 2002 | 0.48 |  |  | 0.04 |  |  |  |  |  |  | -0.13 |  |
| Wessel 2002 |  |  |  | 0.01 |  |  |  |  |  |  |  |  |
| Wessel 2002 |  |  |  | 0.17 |  |  |  |  |  |  |  |  |
| Wessel 2002 |  |  |  | 0.01 |  |  |  |  |  |  |  |  |
| Wessel 2002 |  |  |  | 0.16 |  |  |  |  |  |  |  |  |
| Wessel 2002 |  |  |  | 0.05 |  |  |  |  |  |  |  |  |
| Wessel 2002 |  |  |  | 0.33 |  |  |  |  |  |  |  |  |
| Wessel 2002 |  |  |  | 0.24 |  |  |  |  |  |  |  |  |
| Wessel 2002 |  |  |  | 0.22 |  |  |  |  |  |  |  |  |
| Wessel 2002 |  |  |  | -0.14 |  |  |  |  |  |  |  |  |
| Gorman 2010 | 0.72 |  |  | 0.46 | 0.777 |  |  | 0.44 | 0.776 |  |  |  |
| Gorman 2010 |  |  |  | 0.45 | 0.719 |  |  |  |  |  |  |  |
| Gorman 2010 |  |  |  | 0.42 | 0.767 |  |  |  |  |  |  |  |
| Gorman 2010 |  |  |  | 0.70 | 0.559 |  |  |  |  |  |  |  |
| Gorman 2010 |  |  |  | 0.42 |  |  |  |  |  |  |  |  |
| Gorman 2010 |  |  |  | 0.46 |  |  |  |  |  |  |  |  |
| Silva 2011 | 0.7 |  |  |  |  |  |  |  |  | -0.1 |  |  |
| Swift 2013 | 0.55 | 0.77 |  |  |  |  |  |  |  |  |  |  |
| Swift 2013 | 0.31 | 0.76 |  |  |  |  |  |  |  |  |  |  |
| Swift 2013 | 0.47 |  |  |  |  |  |  |  |  |  |  |  |
| Ladyshewskey 2000 |  |  |  |  |  | 0.85 | 0.84 |  |  |  |  | 1.39 |
| Ladyshewskey 2000 |  |  |  |  |  | 0.79 | 0.63 |  |  |  |  | 1.27 |
| Ladyshewskey 2000 |  |  |  |  |  | 0.87 | 0.74 |  |  |  |  |  |
| Stratford 1990 |  | 0.82 |  |  |  |  |  |  |  |  |  |  |
